# Synthesis, Structure, and Stability of Copper(II) Complexes Containing Imidazoline-Phthalazine Ligands with Potential Anticancer Activity

**DOI:** 10.3390/ph18030375

**Published:** 2025-03-06

**Authors:** Łukasz Balewski, Iwona Inkielewicz-Stępniak, Maria Gdaniec, Katarzyna Turecka, Anna Hering, Anna Ordyszewska, Anita Kornicka

**Affiliations:** 1 Department of Chemical Technology of Drugs, Faculty of Pharmacy, Medical University of Gdansk, Gen. J. Hallera 107, 80-416 Gdańsk, Poland; 2 Department of Pharmaceutical Pathophysiology, Faculty of Pharmacy, Medical University of Gdansk, Gen. Dębinki 7, 80-211 Gdańsk, Poland; 3 Faculty of Chemistry, Adam Mickiewicz University, Uniwersytetu Poznańskiego 8, 61-614 Poznań, Poland; 4 Department of Pharmaceutical Microbiology, Faculty of Pharmacy, Medical University of Gdansk, Gen. J. Hallera 107, 80-416 Gdańsk, Poland; 5 Department of Biology and Pharmaceutical Botany, Faculty of Pharmacy, Medical University of Gdansk, Gen. J. Hallera 107, 80-416 Gdańsk, Poland; 6 Department of Inorganic Chemistry, Faculty of Chemistry and Advanced Materials Centers, Gdańsk University of Technology, Narutowicza 11/12, 80-233 Gdansk, Poland

**Keywords:** synthesis, phthalazin-1(2*H*)-imine, phthalazin-1(2*H*)-one, 4,5-dihydro-1*H*-imidazole, imidazoline, copper(II) complexes, structure, cytotoxic activity, antioxidant properties

## Abstract

**Background/Objectives**: Recently, there has been great interest in metallopharmaceuticals as potential anticancer agents. In this context, presented studies aim to synthesize and evaluate of two copper(II) complexes derived from phthalazine- and imidazoline-based ligands against on three human cancer cell lines: cervix epithelial cell line (HeLa), breast epithelial-like adenocarcinoma (MCF-7), and triple–negative breast epithelial cancer cell line (MDA-MB-231), as well as non-tumorigenic cell line (HDFa). Moreover their antimicrobial, and antioxidant properties were assessed. **Methods**: The synthetized compounds—both free ligands **L1**, **L2**, **L3** and copper(II) complexes **C1** and **C2**—were characterized by elemental analysis, infrared spectroscopy. Additionally, a single-crystal X-ray diffraction studies we performed for free ligand **L3** and its copper(II) complex **C2**. The stability of Cu(II)-complexes **C1** and **C2** was evaluated by UV-Vis spectroscopy. The cytotoxic potency of free ligands and their copper(II) complexes was estimated on HeLa, MCF-7, MDA-MB-231, as well as non-cancerous HDFa by use of an MTT assay after 48 h of incubation. Moreover, the antimicrobial activity of ligands **L1** and **L3** and their copper(II) complexes **C1** and **C2** was evaluated using reference strains of the following bacteria and yeasts: *Staphylococcus aureus*, *Escherichia coli*, and *Candida albicans*. The free radical scavenging properties of free ligands **L1**, **L3** and the corresponding copper(II) complexes **C1**, **C2** was tested with two colorimetric methods—ABTS, DPPH, and reduction ability assay (FRAP). Additionally, the ADME webtool was used to assess the drug-likeness of the synthesized compounds, as well as their physicochemical and pharmacokinetic properties. **Results**: Copper(II) complex **C2** exhibited antitumor properties towards MDA-MB-231 compared with Cisplatin (cancer cell viability rate of 23.6% vs. 22.5%). At a concentration of 200 μg/mL, complexes **C1** and **C2** were less cytotoxic than the reference Cisplatin against a normal, non-cancerous skin fibroblast cell line (HDFa). According to in vitro tests, **C2** reduced the viability of HeLa, MCF-7, and MDA-MB-231 cells by about 57.5–81.2%. It was evident that all compounds were devoid of antibacterial or antifungal activity. In vitro assays revealed that a moderate antiradical effect was observed for free ligand **L1** containing phthalazin-1(2*H*)-imine in the ABTS radical scavenging assay (IC_50_ = 23.63 µg/mL). **Conclusions**: The anticancer studies revealed that the most potent compound was copper(II) complex **C2** bearing a phthalazin-1(2*H*)-one scaffold. None of the tested compounds showed antimicrobial or antifungal activity. This feature seems to be beneficial in terms of their potential uses as anticancer agents in the future. In vitro antiradical assays revealed that a moderate antioxidant effect was observed only for free ligand **L1** containing phthalazin-1(2*H*)-imine.

## 1. Introduction

The presence or absence of three receptors is particularly important when determining the type of breast cancer: the estrogen receptor (ESR1), the progesterone receptor (PGR), and the HER2 epidermal growth factor receptor (ERBB2). Tumors that lack the expression of these three receptors are called triple-negative breast cancer (TNBC) [1,2]. In comparison with other breast cancers, TNBC has particularly high recurrence, metastasis, and mortality rates [3]. As a result of its aggressive nature and lack of targeted therapies, TNBC stands out from other cancers. This type of cancer—typically observed in young women with BRCA1 gene mutations—is the leading cause of mortality in the industrial world and needs multimodal therapeutic schemes.

To date, multiple approaches have attempted to improve the care of patients with TNBC. Among them, chemotherapy is still the most relevant strategy. It includes epidermal growth factor receptor (EGFR)-targeted agents, androgen receptor-targeted agents, and poly (ADP-ribose) polymerase (PARP) inhibitors. It is unfortunate that their use has been restricted to clinical trials, and further work is necessary to identify new targets. Research has indicated that the Hedgehog or Wnt/b-Catenin signaling pathways may serve as promising therapeutic strategies for TNBC treatment [4,5,6]. An acquired resistance of triple-negative breast cancer cells, limited effectiveness of the above-mentioned chemotherapeutic agents, and their significant toxicity prompt the search for novel synthetic compounds with higher potency and selectivity towards tumor cells.

Recently, there has been great interest in metallopharmaceuticals as potential anti-TNBC therapeutics [7,8,9,10,11,12]. Several metal-containing drugs were developed after discovering that *cis*-diaminodichloridoplatinum(II)—well known as Cisplatin—possessed anticancer properties [13]. Nowadays, Cisplatin and its derivatives are frequently used in the treatment of different types of cancer, including TNBC [14,15,16,17,18,19]. As a result of its interaction with purine bases on DNA, Cisplatin produces DNA lesions, activating several signal transduction pathways which trigger the apoptosis response [20]. Aside from the widespread use of Cisplatin as a chemotherapeutic drug, it has several significant drawbacks, including toxic side effects and drug resistance [21].

**Figure 1:**
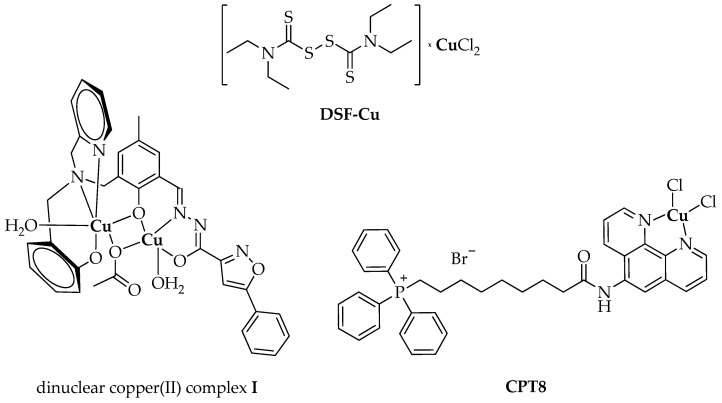
Chemical structures of copper(II) compounds with anti-TNBC activity.

Consequently, there is growing interest in other transition metal complexes with reduced side effects, resistance, and enhanced anticancer activity. Considerable attention has been paid to copper(II) complexes which exhibit pronounced antiproliferative activity [22,23,24,25,26,27]. Their antitumor effect results from various mechanisms, including intercalation and cell cycle arrest, the inhibition of topoisomerases I, II and the perturbation of DNA replication, the induction of apoptosis, and balancing reactive oxygen species (ROS) concentrations [28,29,30,31,32,33,34,35,36,37,38,39]. It was found that the combination of copper(II) salt with Disulfiram (DSF)—a drug used to treat alcoholism with chemosensitizing activity—results in a cytotoxic complex that induces MDA-MB-231 cell death. This complex is highly selective and inhibits proteasomal activity in these cells before inducing apoptosis [40] (**DSF-Cu**, Figure 1). It has been demonstrated that copper(II) dinuclear complexes containing isoxazole-derived aroylhydrazones (copper complex **I** containing 5-phenylisoxazole; Figure 1) interact with calf-thymus DNA exhibiting cytotoxicity towards MDA-MB-231 [41]. In 2008, the cytotoxic effect of copper(II) complexes with diethyldithiocarbamates on MDA-MB-231 cells associated with the inhibition of proteasomes was reported on by Boris Cveck and coworkers [42]. In response to this observation, copper(II) complexes are now commonly studied as potential chemotherapeutic agents for the treatment of TNBC [43,44,45]. Recently, it was demonstrated that the phenanthroline copper(II) complex modified with triphenylphosphonium alkyl chain (**CPT8**, Figure 1) showed an antiproliferative effect towards triple-negative breast cancer. In TNBC cells, **CPT8** leads to the activation of the PINK1/Parkin and BNIP3 pathways, inducing mitophagy [43].

**Figure 2:**
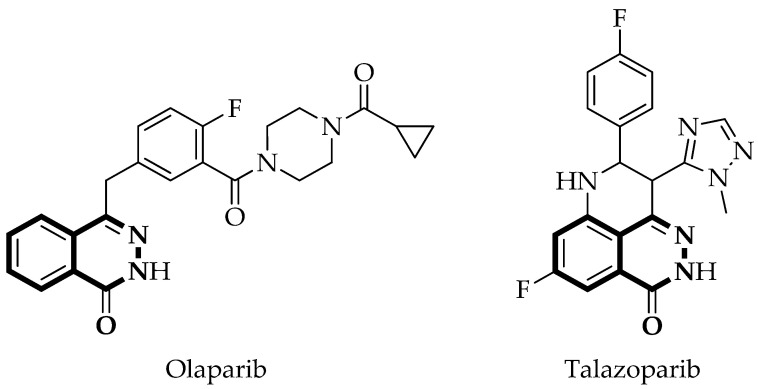
Poly(ADP-ribose) polymerase inhibitors—Olaparib and Talazoparib.

There has been increased interest in compounds containing a central phthalazine skeleton because they exhibit pronounced anticancer properties [46,47,48,49,50]. It is noteworthy that phthalazin-1(2*H*)-one is present in the structure of both Olaparib (Figure 2), a poly(ADP-ribose) polymerase inhibitor used for treating cancers of the ovaries, breasts, and prostate [51], as well as Talazoparib (Figure 2), which is prescribed for advanced breast cancer with germline BRCA mutations [52].

**Figure 3:**
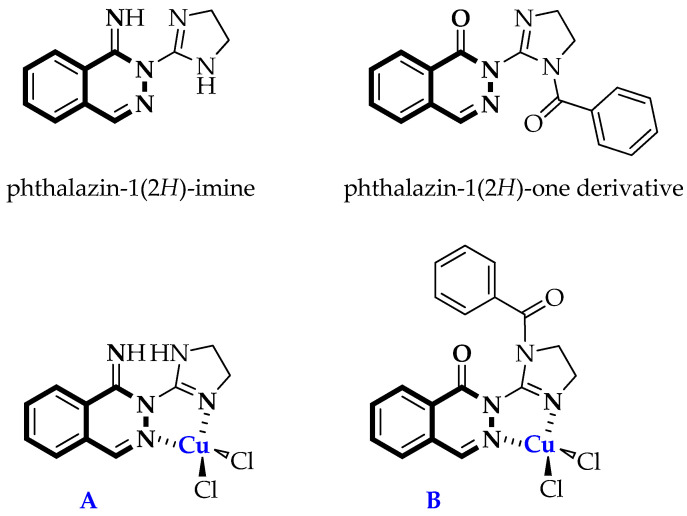
Chemical structures of phthalazine-based ligands: 2-(4,5-dihydro-1*H*-imidazol-2-yl)phthalazin-1(2*H*)-imine, phthalazin-1(2*H*)-one derivative, and their mononuclear copper(II) complexes **A** and **B**.

Our previous studies indicated that newly synthesized copper(II) complexes with benzimidazoles, imidazolidino-2-thiones, 2-iminocoumarins, and isoquinoline possess promising antiproliferative activity against several human tumor cell lines [53,54,55,56]. By continuing our investigations concerning the anticancer activity of copper(II) complexes in this paper, we wish to describe the reactions of phthalazin-1(2*H*)-imine [57] with benzoyl chloride and copper(II) chloride, determine the X-ray structure of the novel phthalazin-1(2*H*)-one-based ligand and its copper(II) complex, and obtain the results of studies on their anticancer, antimicrobial, and antioxidant properties (Figure 3).

## 2. Results and Discussion

### 2.1. Chemistry

**Scheme 1:**
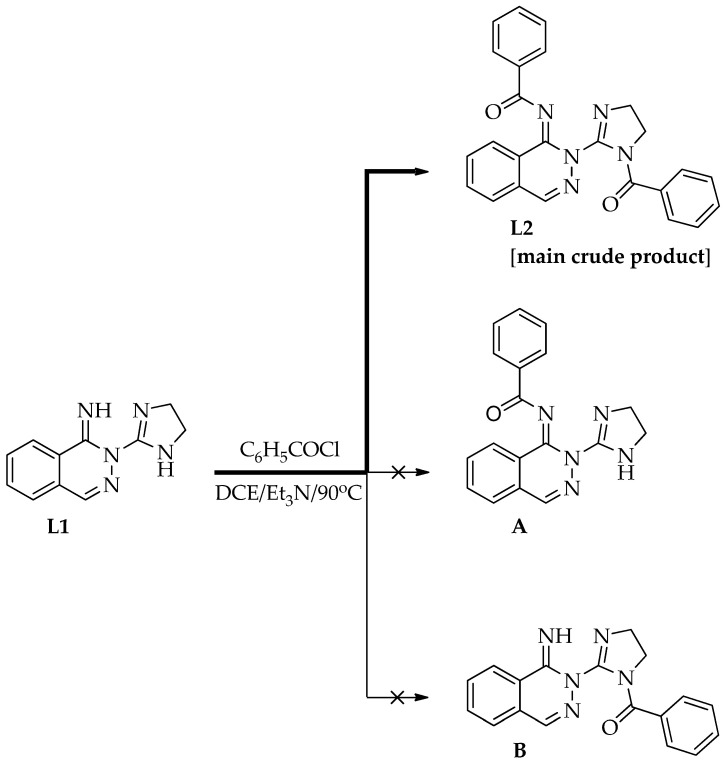
Synthesis of crude *N*-(2-(1-benzoyl-4,5-dihydro-1*H*-imidazol-2-yl)phthalazin-1(2*H*)-ylidene)benzamide (**L2**).

Recently, we have been exploring the transformations of the mentioned 2-(4,5-dihydro-1*H*-imidazol-2-yl)phthalazin-1(2*H*)-imine (ligand **L1**; Scheme 1) (see Supplementary Materials, Figure S1). We assumed that the imine and secondary amine groups in the structure of substrate **L1** were susceptible to reactions with sulfonyl chlorides forming corresponding sulfonamides [57,58]. As part of the present work conducted in our laboratory, we present the results of the reactions between 2-(4,5-dihydro-1*H*-imidazol-2-yl)phthalazin-1(2*H*)-imine (**L1**) and acyl chlorides to produce analogous *N*-(2-(1-aroyl-4,5-dihydro-1*H*-imidazol-2-yl)phthalazin-1(2*H*)-ylidene)benzamides.

Upon the treatment of compound **L1** with two-fold molar excess of benzoyl chloride, the corresponding di-substituted *N*-(2-(1-benzoyl-4,5-dihydro-1*H*-imidazol-2-yl)phthalazin-1(2*H*)-ylidene)benzamide **L2** was formed (Scheme 1) (see Supplementary Materials, Figures S2–S5). The reaction was carried out in anhydrous dichloroethane (DCE) at a temperature of 90 °C. Progress was controlled by TLC. Our attempts to obtain mono-*N*-substituted main products failed, e.g., *N*-(2-(4,5-dihydro-1*H*-imidazol-2-yl)phthalazin-1(2*H*)-ylidene)benzamide (**A**) or (2-(1-iminophthalazin-2(1*H*)-yl)-4,5-dihydro-1*H*-imidazol-1-yl)(phenyl)methanone (**B**). We observed the formation of compound **L2** (di-substituted main product) even at a lower rage of temperatures (20–50 °C) and in a stoichiometric ratio (1:1) of compound **L1** and benzoyl chloride (Scheme 1).

**Scheme 2:**
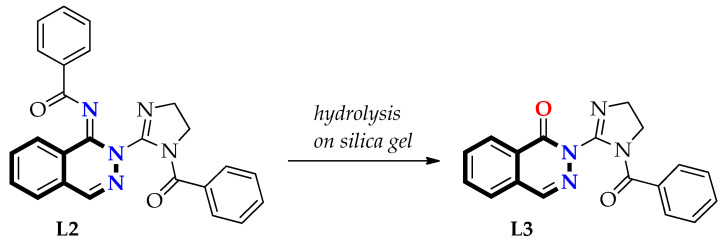
Transformation of phthalazin-1(2*H*)-imine derivative **L2** into phthalazin-1(2*H*)-one-based ligand **L3**.

During our experimental research, we unexpectedly observed the formation of phthalazin-1(2*H*)-one derivative **L3** as a novel lower-mass product (Scheme 2) (see Supplementary Materials, Figures S6–S9). The purification of crude *N*-(2-(1-benzoyl-4,5-dihydro-1*H*-imidazol-2-yl)phthalazin-1(2*H*)-ylidene)benzamide (**L2**) by a preparative, centrifugally accelerated thin-layer chromatograph (chromatotron) or classical column chromatography resulted in the hydrolysis of a phthalazin-1(2*H*)-imine ring due to the acidic character of silica gel and the presence of water.

**Scheme 3:**
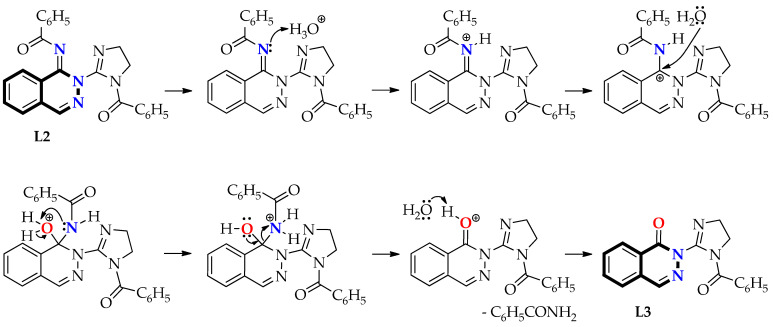
Proposed mechanism of hydrolysis of ligand **L2** upon purification on silica gel.

Compounds bearing an imine moiety are well understood in terms of their chemical stability and behavior [59,60]. The proposed mechanism of the formation of 2-(1-benzoyl-4,5-dihydro-1*H*-imidazol-2-yl)phthalazin-1(2*H*)-one (**L3**) is shown in Scheme 3. The protonation (the pH value of silica gel was about 6.7–6.8) of the nitrogen atom (C=N) gives an electrophilic iminium ion which is attacked by a water molecule. The next step is the transfer of the proton from the oxygen atom to nitrogen. This process gives an intermediate. Then, the deprotonation of (2-(1-benzoyl-4,5-dihydro-1*H*-imidazol-2-yl)phthalazin-1(2*H*)-ylidene)oxonium yields the phthalazin-1(2*H*)-one-based ligand L3 (Scheme 3).

The structure of ligand **L3** was determined by collected spectroscopic data (IR, ^1^H-NMR, ^13^C-NMR, MS) as well as an elemental analysis (see Supplementary Materials, Figures S6–S9). In the spectroscopic characterization of isolated product **L3**, we noted strong bands at 1678 cm^−1^ and 1660 cm^−1^ attributed to two C=O stretching frequencies of phthalazin-1(2*H*)-one skeleton and benzamide moiety. Additionally, aromatic or heteroaromatic skeletal and C=N moiety stretching vibrations were observed in a range of 1578 to 1634 cm^−1^ (see Supplementary Materials, Figure S6). The proton magnetic spectrum (^1^H-NMR) registered in DMSO-*d_6_* (400 MHz) at 20–22 °C revealed a singlet attributable to proton C4-H of phthalazin-1(2*H*)-one at 8.40 ppm and two multiplets in the ranges *δ =* 3.97–4.02 ppm and *δ =* 4.15–4.19 ppm—noting the integration for four protons—which can be attributed to the CH_2_-CH_2_ bridge of the 4,5-dihydro-1*H*-imidazole ring. Nine heteroaromatic protons appear in the spectrum as four multiplet signals in the range of 6.96–7.92 ppm and doublet (*J* = 7.8 Hz) at 8.00 ppm, respectively (see Supplementary Materials, Figure S7). The ^13^C-NMR spectrum of compound **L3** recorded in DMSO-*d_6_* (100 MHz) solution at 20–22 °C revealed signals at 48.93 ppm and 50.85 ppm—showing carbon atom C4′ and carbon atom C5′ of the 4,5-dihydro-1*H*-imidazole ring. In the range of 126.19 ppm to 140.50 ppm, thirteen aromatic carbon atoms of the phthalazin-1(2*H*)-one system and phenyl ring were observed. The quaternary C2′ carbon atom of 4,5-dihydro-1*H*-imidazole appeared at 151.03 ppm, whereas signals at 158.46 ppm and 167.46 ppm can be assigned to two carbonyl carbon atoms (C=O) of the C1 carbon atom of phthalazin-1(2*H*)-one and the benzoyl moiety (see Supplementary Materials, Figure S8). The electrospray ionization mass spectrum pattern indicated the presence of protonated ligand **L3**, *m*/*z* = 319 [M + H]^+^, and its adducts with sodium ion and acetonitrile molecule, *m*/*z* = 341 [M + Na]^+^ and *m*/*z* = 382 [M + Na + MeCN], respectively (see Supplementary Materials, Figure S9).

#### Synthesis of Dichloro[2-(4,5-dihydro-1*H*-imidazol-2-yl)phthalazin-1(2*H*)-imine]copper(II) (**C1**) and Dichloro[2-(1-benzoyl-4,5-dihydro-1*H*-imidazol-2-yl)phthalazin-1(2*H*)-one]copper(II) (**C2**)

Bearing in mind that the incorporation of metal ions into organic compounds is beneficial in terms of anticancer activity and cancer cell-oriented selectivity, we focused on the synthesis of complexes of designed ligands **L1**-**L3** with copper [61,62]. Moreover, it was established that the incorporation of copper ions into an organic ligand may reduce toxicity against normal human cells [63] or selectively increase their uptake by cancer cells [64]. Our effort to obtain a coordination compound in the reaction of designed di-substituted ligand **L2** with copper(II) salt failed. This behavior is possible because of the steric hindrance of two benzoyl groups in its structure. Moreover, the stability studies performed for ligand **L2** showed that the absorbance of the solution was decreased. Additionally, we observed some isosbestic points in the UV-VIS spectra of **L2**. This may suggest a possible reaction in the buffer solution.

**Scheme 4:**
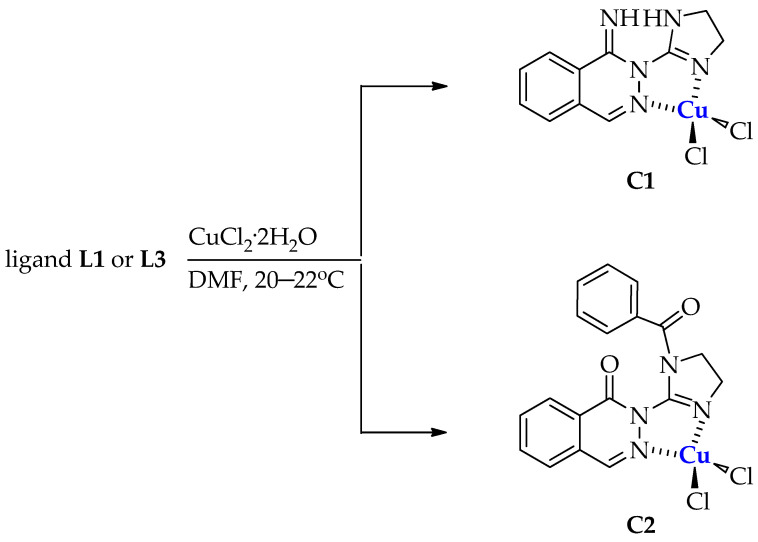
Synthesis of dichloro[2-(4,5-dihydro-1*H*-imidazol-2-yl)phthalazin-1(2*H*)-imine]copper(II) (**C1**) and dichloro[2-(1-benzoyl-4,5-dihydro-1*H*-imidazol-2-yl)phthalazin-1(2*H*)-one]copper(II) (**C2**).

Therefore, in this work, we carried out a successful complexation reaction of two ligands: 2-(4,5-dihydro-1*H*-imidazol-2-yl)phthalazin-1(2*H*)-imine (**L1**) and 2-(1-benzoyl-4,5-dihydro-1*H*-imidazol-2-yl)phthalazin-1(2*H*)-one (**L3**). Due to the presence of two nitrogen atoms capable of coordinating a transition metal in their structures, these ligands were subjected to a reaction with copper(II) chloride in 98% dimethylformamide at room temperature (Scheme 4).

Dichloro[2-(4,5-dihydro-1*H*-imidazol-2-yl)phthalazin-1(2*H*)-imine]copper(II) (**C1**) precipitated out of the solution immediately upon the addition of copper(II) chloride dihydrate as an intense green powder. Attempts were made to obtain single crystals of complex **C1** suitable for X-ray analysis by carrying out the reaction at an elevated temperature, gradually adding copper(II) salt as a solution, or changing the solvent (ethanol, *n*-butanol). In each case, the copper(II) complex precipitated as a fine solid. On the other hand, monocrystals of complex **C2** suitable for crystallographic studies were collected by slow evaporation of the solvent at room temperature (20–22 °C) over 7 days.

### 2.2. Structure of Copper(II) Complexes ***C1*** and ***C2***

**Figure 4:**
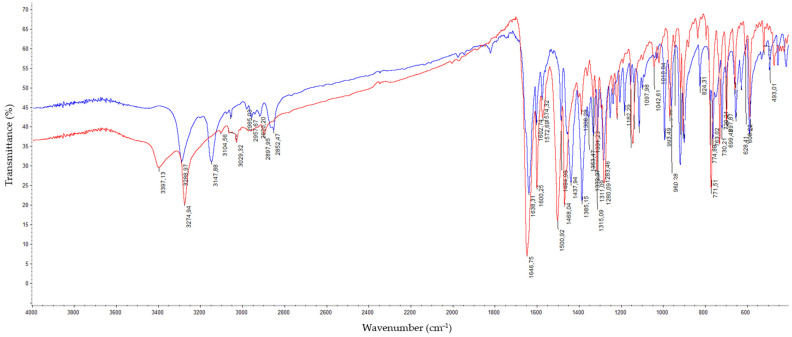
Infrared spectra of **L1** (blue line) and its complex, **C1** (red line).

**Figure 5:**
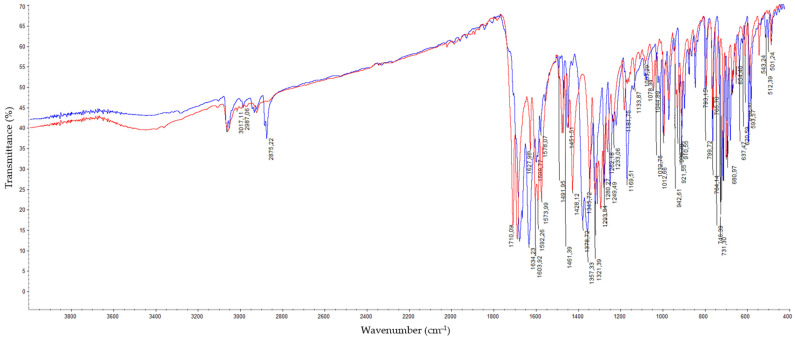
Infrared spectra of **L3** (blue line) and its complex, **C2** (red line).

Copper(II) complexes **C1** and **C2** gave satisfactory elemental analysis results. All were found to have a formula of mononuclear complexes, CuLCl_2_. Transient metal complexes—such as copper(II) complexes—can be evaluated using an infrared spectrum. We compared the IR spectra obtained after the coordination of metal ions with the corresponding IR spectra of free ligands (see Supplementary Materials, IR spectra of free ligands **L1** and **L3**—Figures S1 and S6; IR spectra of copper(II) complexes **C1** and **C2**—Figures S10 and S11). It should be noted that these IR spectra displayed some shifts in absorption bands. This may confirm the chelation of copper(II) ions to the free ligand. The IR spectra of **C1** and **C2** show intense bands above 1600 cm^−1^ that may be attributed to the *ν*(C=N)_phthalazine_, *ν*(C=N)_imidazoline_, and aromatic skeletal (C$\overset{\ldots }{—}$C) modes. Upon complexation, the bands that correspond to the C=N vibrations (in complexes **C1** and **C2**) and carbonyl groups (in complex **C2**) slightly shifted to higher values compared to the corresponding absorptions in free ligands **L1** and **L3**. For example, an increase in the wavenumber 1646 cm^−1^ in **C1** with respect to that of the band at 1638 cm^−1^ in free ligand **L1** was observed (Figure 4). Similarly, 1678 cm^−1^ and 1634 cm^−1^ in ligand L3 shifted to the values 1710 cm^−1^ and 1689 cm^−1^ in the corresponding copper(II) complex **C2**, respectively (Figure 5).

In most cases, ligands donate nonbonding electrons (σ-electron density) to metals while accepting electron density (π-electron density) from them through the overlap of t_2g_ orbitals between the metal and ligand. Thus, ligands may act both as a σ-donors and π-acceptors, and this process is called π-back-bonding [65]. On the other hand, electrons from transient metal ions are donated to the ligand π* orbital, adding electron density to the ligand antibonding molecular orbital. It leads to the weakening of the bond, which is observed as lengthening of the bond and lowering of the infrared stretching frequency. Contrary to this observation, in the cases of studied complexes **C1** and **C2**, the coordination of copper(II) ions increased the C=N double-bond character and increased the IR frequencies of this group. This result cannot be clearly explained. Copper(II) ion chelation may lead to the stabilization of the C=N bond in copper(II) complexes **C1** and **C2**. This may be attributed to changes in the specific conformation of the ligand molecules, leading to wider bending angles at the carbon and nitrogen atoms.

Since copper(II) complexes display distinct magnetic properties, their proton nuclear magnetic resonance spectra (^1^H-NMR) cannot be registered [66]. In light of this, we sought to achieve single crystals of both the ligand and its corresponding copper(II) complex suitable for X-ray analysis.

#### Crystallographic Studies of Ligand **L3** and Copper(II) Complex **C2**

**Figure 6:**
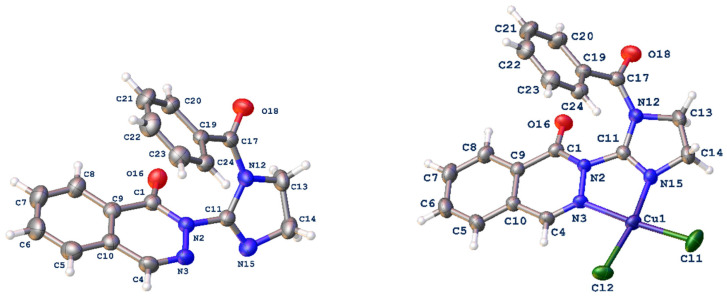
Molecular structure of **L3** (left) and **C2** (right). Displacement ellipsoids are shown at 50% probability level.

The molecular structures of the 1-phthalazinone derivative **L3** and its complex with CuCl_2_, **C2**, are shown in Figure 6. Crystal data and details of the structure refinement of ligand **L3** and copper(II) complex **C2** are collected in Table 1. In the free ligand, the imidazoline and phthalazine fragments are strongly twisted around the N2-C11 bond, as shown by the torsion angle N3-N2-C11-N15 of 55.6°. The tertiary amide group, which is in the *Z* configuration, is twisted around the amidic C17-N12 bond (C19-C17-N12-C11 torsion angle of 36.1°) and highly nonplanar. The deviation of N12 from the plane formed by the three attached C atoms is 0.139 Å. The planes of the phenyl group and the pyridazine ring form a dihedral angle of 34.98°, and the ring centroids are separated by 3.734 Å. Most likely, the adopted molecular conformation of L3 results, to some degree, from an attractive intramolecular interaction of the two carbonyl groups, which form a relatively close O16···C17 contact of 2.955 Å (the sum of van der Waals radii 3.22 Å). Attractive intra- and intermolecular carbonyl–carbonyl interaction is well known to occur [67,68,69,70]. It has been shown that it can influence the geometries and functions of molecules with multiple carbonyl groups [69,70]. In **L3**, the two carbonyl groups, separated by four bonds, form one short O···C contact, and the torsion angle T_C=O···C=O_ is about 180°, while the angle θ_O···C=O_ is close to 109.5° (for the exact values see Table 2), which means that their mutual arrangement can be classified as motif III [67,69]. The molecule of **L3**, in the conformation observed in the solid state, is pre-organized to coordinate metals via the N3 phthalazine and N15 imidazoline atoms to form a five-membered chelate ring. As evidenced by the crystal structure of **C2**, the binding of the Cu^2+^ ion strongly influences the ligand conformation and the carbonyl–carbonyl interaction. While the changes in the conformation of the 1-benzoylimidazoline fragment are rather small, the torsion angle about the N2-C11 bond between the rings is significantly reduced (N3-N2-C11-N15 14.30°). Metal ion binding leads to a redistribution of the electron density in the ligand, which is reflected in the significant lengthening of the N15-C11, N12-C17, and N2-C1 bonds and the shortening of the C11-N12 and C11-N2 bonds (Table 3). Most importantly, the O16···C17 contact between the carbonyl groups in the bound ligand is reduced to 2.655 Å, i.e., it is shortened by 0.3 Å compared to the free ligand. The geometric parameters describing the C=O···C=O interaction in the free and bound ligand are given in Table 2. In **C2**, the coordination environment of the Cu^2+^ ion can be described as distorted square planar, with the Cu-Cl and Cu-N bonds having average lengths of 2.2341(5) Å and 2.024 (2) Å, respectively. However, there is an additional Cu-Cl contact of 2.8849(6) Å between the inversion center-related molecules that assembles the monomeric molecules into a dinuclear complex with a {Cu_2_(μ_2_-Cl)_2_} core in which the Cu···Cu distance is 3.5950(5) Å. In this dinuclear complex, the coordination geometry of the Cu(II) atom (N_2_Cl_3_) can be described as distorted square pyramidal (see also CheckCIF/PLATON reports of ligand L3 and copper(II) complex **C2** in Figures S12 and S13 in Supplementary Materials, pages 9–10).

### 2.3. Stability of Copper(II) Complexes—UV-Vis Investigations of Compounds ***C1*** and ***C2*** in Phosphate-Buffered Saline Solution (PBS)

**Figure 7:**
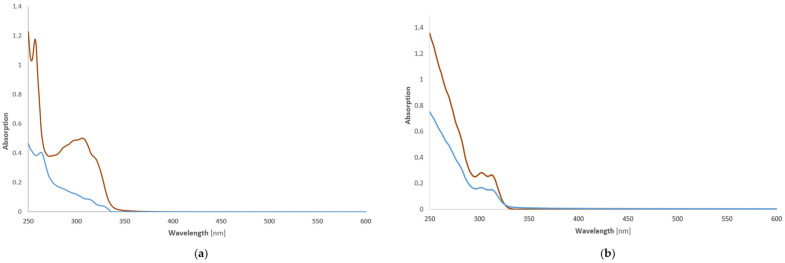
UV-Vis spectra of ligands **L1** and **L3** (orange lines) and copper(II) complexes **C1** and **C2** (blue lines) in 10% DMSO/PBS (pH 7.4) at concentration of 0.10 mM at 20–22 °C: (**a**) **L1** and **C1** and (**b**) **L3** and **C2**.

**Figure 8:**
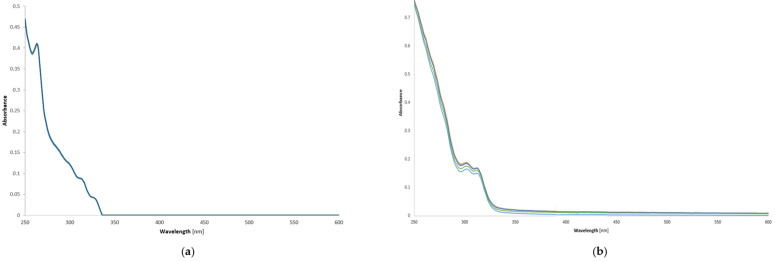
Time-dependent UV-Vis spectra of copper complexes(II) **C1** (**a**) and **C2** (**b**) in PBS (pH 7.4) at concentration of 40 µM at 20–22 °C. Spectra were recorded at t = 0, 2, 4, 6, 8, 10, and 12 h after addition of tested compounds to buffer (each spectrum recorded has a different color).

The stability of copper(II) complexes **C1** and **C2** was determined in phosphate-buffered solution at 20–22 °C (pH 7.4) by using a UV-vis spectrophotometer. We recorded UV-vis spectra every 120 min over the 12 h incubation period in order to determine whether the spectra changed over time. Figure 7 shows the UV-Vis spectra of free ligands **L1** and **L3** overlapped on the spectra of the corresponding copper(II) complexes C1 and **C2** dissolved in 10% DMSO/PBS at a concentration of 0.10 mM. The time-dependent changes in the UV-Vis spectra of **C1** and **C2** at a temperature in the range of 20–22 °C over 12 h of incubation in PBS are presented in Figure 8. Tested complexes **C1** and **C2** showed no noticeable changes in their spectra over a period of 12 h of incubation.

### 2.4. In Silico Molecular Modeling of Ligand ***L3***

**Figure 9:**
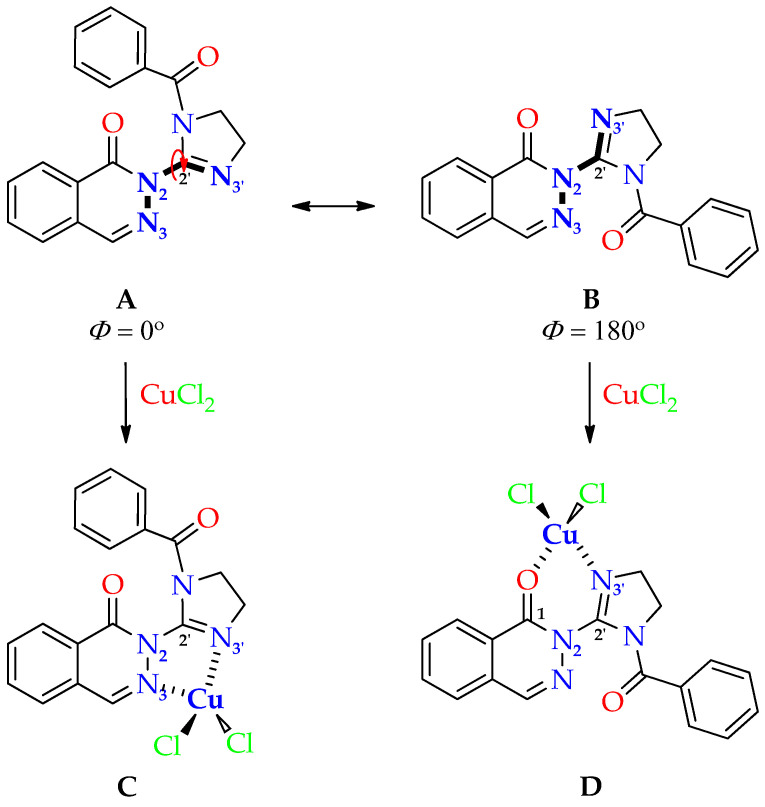
Structures of conformers (**A**,**B**) of ligand **L3** and corresponding copper(II) complexes (**C**,**D**).

In order to assess the relative stability of rotamers **A** and **B** of ligand **L3** used for the reaction with copper(II) chloride, a conformational analysis was carried out at the ab initio level. To perform calculations, we used the density functional (B3LYP) method with the 6-31G** basis set implemented into the SPARTAN program [71]. These two conformers, **A** and **B**, may form two possible corresponding copper(II) complexes, namely one with a five-membered -Cu-N_3_-N_2_-C_2′_-N_3_- chelate ring (structure **C**) or one involving the carbonyl group of the phthalazin-1(2*H*)-one moiety with a six-membered ring -Cu-O-C_1_-N_2_-C_2′_-N_3_- (structure **D**) (Figure 9).

The obtained data reveal that conformer **B** with torsion angle N_3_-N_2_-C_2′_-N_3′_ *Φ* = 180° was found to be lower in energy (E = −667,283.62055 kcal/mol) than molecule **A** (E = −667,276.780695 kcal/mol) with torsion angle N_3_-N_2_-C_2′_-N_3_ *Φ* = 0° (ΔE = 6.839855 kcal/mol). In addition, based on dipole moments (calculated μ), conformer **B** (μ = 3.71 D) was predicted to prevail over **A** (μ = 1.47 D) in a polar solvent (DMF). It should be concluded that upon chelation in DMF solution, the more stable conformer, **B** (*Φ* = 180°), forms a mononuclear copper(II) complex (molecule **C**), where the central copper ion is coordinated by the following ligand: 2-(1-benzoyl-4,5-dihydro-1*H*-imidazol-2-yl)phthalazin-1(2*H*)-one (**L3**). The formation of a five-membered ring involves two nitrogen atoms—at position N_3_ of the phthalazin-1(2*H*)-one system and position N_3′_ of the imidazoline ring (Figure 9).

Regarding the above, it is worth noting that the conformation of molecule **B** is more stable than conformer **A**. This conformer may predominate in the solvent used for the reaction. We can assume that in conformer **A**, with a torsion angle *Φ* = 0°, the localization of the two nitrogen atoms is beneficial for binding copper(II) ions. Therefore, the formation of a five-membered chelate ring requires a certain amount of energy, which is compensated for by novel coordinate bonds between copper ions and nitrogen atoms: Cu^2+…^N_3_=N_2_ and Cu^2+…^N_3′_=C.

**Figure 10:**
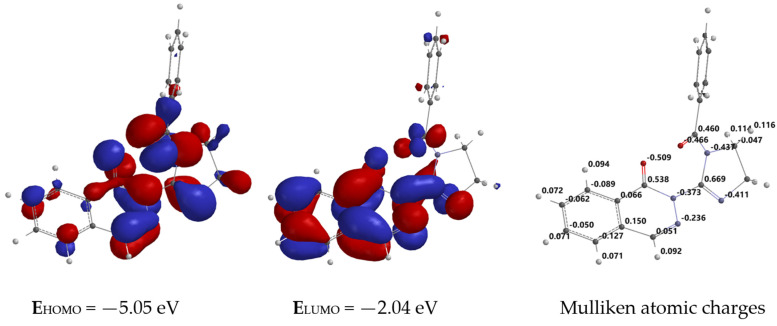
Orbital diagrams of HOMO and LUMO, their energies (E, eV), and Mulliken atomic charge distribution for optimized structure of ligand **L3**.

In turn, to determine the reactivity of ligand **L3** towards bonding with transient metals and identify reactive sites, the electronic structure of **L3** was computed using the ab intio DFT method at the B3LYP/6-31G** basis set (SPARTAN). Figure 10 shows the distribution and contours of the frontier orbitals: the highest occupied molecular orbital, HOMO; the lowest unoccupied molecular orbital, LUMO; and the Mulliken atomic charge localization over **L3** molecule atoms in a gas phase. The highest occupied molecular orbital is limited to the carbon C_4_ and nitrogen N_3_ atoms of the phthalazin-1(2*H*)-one system and the nitrogen atom N_3′_ of the imidazoline ring (E_HOMO_ = −5.05 eV), while the lowest unoccupied molecular orbital (LUMO) is located mostly on the entire phthalazin-1(2*H*)-one scaffold (E_LUMO_ = −2.04 eV). The calculated HOMO-LUMO energy gap (E_g_ = 3.01 eV) may suggest high reactivity of ligand **L3** towards bonding with transient metals.

The Mulliken population analysis of ligand **L3** shows a net of atomic charges directly related to molecular properties. We can distinguish the most shielded by electron cloud atoms: two oxygen atoms of carbonyl groups (C=O) with negative charges of −0.509 and −0.466 and two nitrogen atoms bonded to an electrophilic carbon atom C_2′_ of the 4,5-dihydro-1*H*-imidazole ring (negative charges of −0.437 and −0.411) (Figure 10) [71].

### 2.5. In Vitro Anticancer Potential

In view of the fact that metal-coordinated compounds have increased biological activity when compared to free ligands, we tested both free ligands and copper complexes for their anticancer activity. It should be pointed out that upon complexation, selectivity towards cancer cells can be improved [72,73].

The anticancer potency of ligands **L1** and **L3** and their copper(II) compounds **C1** and **C2** was evaluated towards human cancer lines, namely the cervix epithelial cell line (HeLa), breast epithelial-like adenocarcinoma (Michigan Cancer Foundation-7 Cells, MCF-7), and breast epithelial cancer cell line (MDA-MB-231) by use of the MTT test after 48 h of incubation. Additionally, a normal, non-cancerous adult human dermal (skin) fibroblast cell line (HDFa) was tested to determine the selectivity towards cancer cell lines. The results of our biological in vitro test are presented in Table 4, Table 5 and Table 6. The effect of ligands **L1** and **L3** and complexes **C1** and **C2** on the viability of cancer cells was measured after 48 h of incubation by an MTT assay. This method measures the metabolic activity of living cells by assessing the reduction in membrane-permeable yellow 3-(4,5-dimethyl-2-thiazolyl)-2,5-diphenyl-2*H*-tetrazolium bromide to dark blue MTT formazan crystals by mitochondrial dehydrogenases. The collected data are the mean of three independent experiments.

From the results obtained for free ligands **L1** and **L3**, it is apparent that only **L1** containing the phthalazin-1(2*H*)-imine skeleton displays low anticancer activity on all three cancers: MDA-MB-231, HeLa, and MCF-7. Additionally, compound **L1** does not affect the non-tumorigenic cell line (HDFa), demonstrating its selectivity towards cancer cells. In contrast to the ligand bearing an imine moiety, the ligand containing carbonyl group **L3** was inactive and did not show antitumor activity against all studied tumor cell lines in a concentration of 200 μg/mL. For this reason, its effect on the normal cell line HDFa was not tested.

Generally, copper(II) complexes **C1** and **C2** show higher anticancer potential than their parent ligands **L1** and **L3**, and the triple-negative breast cancer line was the most sensitive to the tested compounds in the 48 h MTT assay. According to these findings, metal incorporation may enhance the anticancer potency of organic compounds.

**Figure 11:**
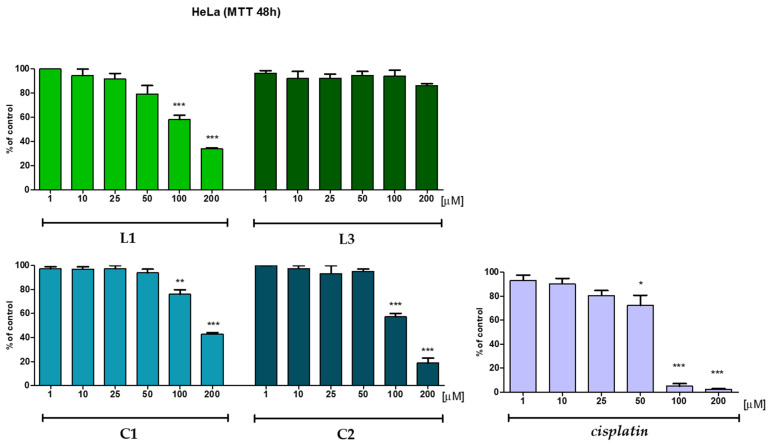
The effect of ligands **L1** and **L3** and complexes **C1** and **C2** on the viability of cervical cancer cells in the HeLa line after 48 h of incubation. An MTT assay was used for measuring cell viability. The data are shown as the mean of three independent experiments * *p* < 0.05; ** *p* < 0.01; *** *p* < 0.001.

**Figure 12:**
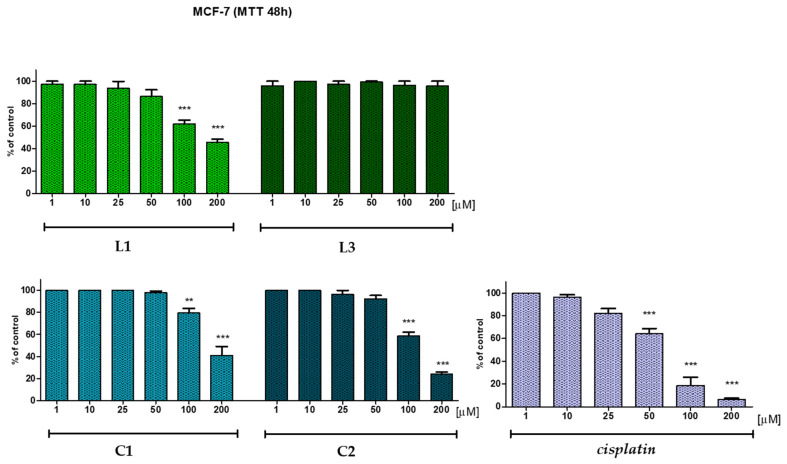
The effect of ligands **L1** and **L3** and complexes **C1** and **C2** on the viability of human breast epithelial-like adenocarcinoma, line MCF-7, after 48 h of incubation. An MTT assay was used for measuring cell viability. The data are shown as the mean of three independent experiments ** *p* < 0.01; *** *p* < 0.001.

**Figure 13:**
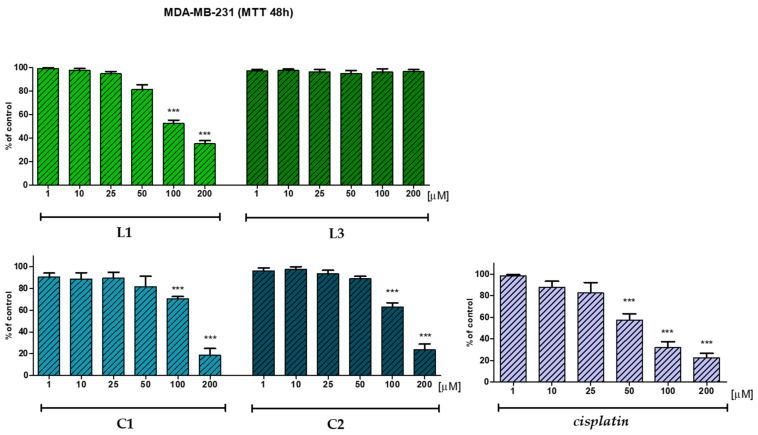
The effect of ligands **L1** and **L3** and complexes **C1** and **C2** on the viability of triple-negative breast cancer cells, line MDA-MB-231, after 48 h of incubation. An MTT assay was used for measuring cell viability. The data are shown as the means of three independent experiments: *** *p* < 0.001.

It is worth noting that in HeLa, MCF-7, and MDA-MB-231, treatment with 100 µg/mL of copper(II) complex **C1** bearing an imine moiety produced no significant anticancer effect (80.5–91.0% of cell viability). The incubation of **C1** decreased cancer cell viability only by about 9–19.5% as opposed to the control. The tested Cu(II) compound **C1** at a concentration of 100 μg/mL showed slightly weaker activity compared to the corresponding free ligand **L1** (52.7–62.1%). The results summarized in Figure 11, Figure 12 and Figure 13 and Table 4, Table 5 and Table 6 show that **C1** proved to be more potent at a concentration of 200 μg/mL (form 18.7% towards the MDA-MB-231 cell line to 56.4% for the HeLa cell line). Thus, Cu(II) complex **C1** exhibited moderate antitumor properties and decreased cancer cells’ viability at a higher concentration. In HeLa cells, compound **C1** decreased their viability by about 43.6% when added at a concentration of 200 μg/mL (Figure 11, Table 4). Similar results were obtained in the human breast epithelial-like adenocarcinoma line MCF-7 when treated with 200 μg/mL of **C1**, which possesses slightly lower potency (49.2%) in reference to free ligand **L1** (45.7%) (Figure 12, Table 5).

On the other hand, triple-negative breast cancer (MDA-MB-231) treatment with copper(II) complex **C1** at a concentration of 200 μg/mL induced significant effects on cell viability (decrease of about 81.3%). Its effectiveness was higher than free ligand **L1** (35.2%) and comparable to copper(II) complex **C2** containing a phthalazin-1(2*H*)-one skeleton (23.6%) (Figure 13, Table 6).

Among all tested compounds, copper(II) complex **C2** bearing a phthalazin-1(2*H*)-one scaffold displayed the best potency towards the MDA-MB-231, HeLa, and MCF-7 cancer cell lines. Compound **C2** showed high growth inhibitory potency against all cancer cell lines in vitro. Cell viability after 48 h of incubation with **C2** at a concentration of 200 μg/mL was in the range of 18.8% to 42.5%. The MTT results showed a higher decrease in cell viability after 48 h of incubation for complex **C2** on the viability of HeLa and MCF-7 compared with **C1** (18.8% for HeLa in contrast to 56.4% and 42.5% for MCF-7 in contrast to 49.2%). Thus, the introduction of a phthalazin-1(2*H*)-one moiety or the presence of a benzoyl substituent may be beneficial for the potency of this class of compounds. Cu(II) complexes **C1** and **C2** at a concentration of 200 μg/mL have shown similar anticancer potency towards the MDA-MB-231 cell line (**C1**: 18.7%; **C2**: 23.6%) in comparison to the reference Cisplatin (22.6%).

**Figure 14:**
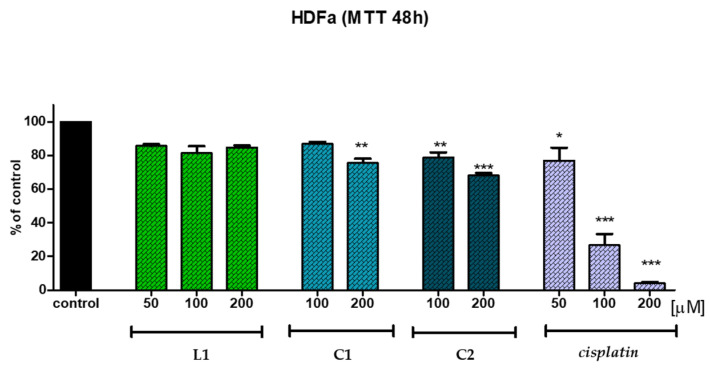
Effect of ligand **L1** and complexes **C1** and **C2** on viability of skin fibroblasts, HDFa line, after 48 h of incubation. MTT assay was used for measuring cell viability. Data are shown as mean of three independent experiments: * *p* < 0.05, ** *p* < 0.01, and *** *p* < 0.001 compared to control.

A non-tumorigenic skin fibroblast cell line (HDFa) is not affected by copper(II) complexes **C1** and **C2** nor, as mentioned above, by free ligand **L1** (Figure 14, Table 7). Although the tested compounds do not demonstrate stronger activity than the reference Cisplatin at a concentration of 200 μg/mL, they are not as cytotoxic to the normal, non–cancerous cell line HDFa (viability in the range of 68.1–84.8% vs. 4.1% for Cisplatin). This is particularly significant since our goal was to develop a lead compound that could be used in the treatment of metastatic triple-negative breast cancer and be more effective, selective, and safe for patients. There is a great deal of difficulty in designing drugs for this type of cancer.

Generally, one may conclude that ligand **L1** was less active than its corresponding metal complex **C1** (34.1–45.7% vs. 18.7–56.4%). Copper(II) complex **C2** exhibited antitumor potential compared with the frequently used anticancer agent Cisplatin (23.6–42.5% vs. 2.5–22.5%). Moreover, Cu(II) complex **C2** at a concentration of 200 μg/mL demonstrated comparable cytotoxic potency against MDA-MB-231 cells compared to Cisplatin (23.6% vs. 22.6%). Due to the lack of potential anticancer activity, free ligand **L3** was not tested on the non-tumorigenic HDFa cell line (94.1–96.5% of cell viability at a concentration of 100 μg/mL and 86.4–96.7% at a concentration of 200 μg/mL, respectively).

These promising preliminary results are the starting point for further biological studies on the anticancer potential of newly designed copper(II) complexes. Overall, these results are consistent with the hypothesis that incorporating metal ions into organic ligands may enhance their cytotoxic effect against cancer cells.

### 2.6. Antimicrobial Activity

The in vitro antimicrobial properties of free ligands **L1** and **L3** and their Cu(II) complexes **C1** and **C2** were investigated on representative Gram-positive and Gram-negative bacterial reference strains of bacteria, namely *Staphylococcus aureus* strain ATCC 6538, *Escherichia coli* strain ATCC 8739, as well as a strain of *Candida albicans* yeast, ATCC 102231. The antimicrobial properties were expressed as MIC values (minimum inhibitory concentration) and MBC values (minimum bactericidal concentration) in micrograms per milliliter [µg/mL]. The MIC and MBC values of the tested compounds were determined by the microbroth dilution method according to the EUCAST (European Committee on Antimicrobial Susceptibility Testing) [74,75]. The obtained results of the MIC and MBC of tested compounds **L1**, **L3**, **C1**, and **C2** on reference strains of bacteria and yeasts are included in the Supplementary Materials (Table S1).

As revealed by the data in Table S1 (Supplementary Materials, page 11), both ligands **L1** and **L3** and Cu(II) compounds **C1** and **C2** displayed no antimicrobial activity against representative strains of Gram-positive bacteria, Gram-negative bacteria, and yeasts. In light of the anticancer activity of copper(II) complex **C2**, it is important to note that this compound does not exhibit bioactivity towards microorganisms, thereby causing no harm to the human microbiota during treatment. It is noteworthy that microbes in the host gastrointestinal tract (GI tract, gut tract) contribute significantly to physiology and health [76]. Through the colonization of mucosal membranes and the production of different antimicrobial substances, gut microbiota protect the host from other pathogens. They play a significant role in digestion and metabolism [77]. Through GI–brain communication, gut flora may impact the mental and neurological functions of the host [78,79,80]. Finally, gut microorganisms enhance the host’s immune system [81].

### 2.7. Determination of Antioxidant Activity

Anticancer agents that display additional antiradical activity are generally very effective. In comparison to conventional chemotherapeutics, they are associated with fewer side effects. This makes antioxidants a promising option for treating many cancers, specifically TNBC [82,83,84,85,86]. It is well known that many types of cancer are caused by an imbalance between reactive oxygen species (ROS) and the antioxidant system. Therefore, increasing the antioxidant capacity of cancer cells is one method of reducing tumor growth. On the other hand, compounds displaying pro-oxidant properties have been reported to induce DNA damage responses, leading to cell death [87,88]. For that reason, we decided to evaluate the antiradical properties of free ligands **L1** and **L3**, as well as copper(II) complexes **C1** and **C2**.

The free radical scavenging properties of free ligands **L1** and **L3** and the corresponding copper(II) complexes **C1** and **C2** were tested with colorimetric methods—namely ABTS utilizing 2,2′-azinobis(3-ethylbenzothiazoline-6-sulphonic acid), DPPH with 2,2′-diphenyl-1-picrylhydrazyl, and a reduction ability assay (ferric ion-reducing antioxidant power test, FRAP). L-ascorbic acid was used as a reference compound (positive control). These simple colorimetric tests provide repeatable results. The collected data are expressed as the concentration of tested compound that results in 50% of the maximum scavenging activity (IC_50_ values; Table 8).

From our collected data in Table 8, it can be concluded that only free ligand **L1** containing a phthalazin-1(2*H*)-imine scaffold exhibited good scavenging ability in the ABTS test compared with ascorbic acid (IC_50_ = 23.63 µg/mL vs. IC_50_ = 15.68 µg/mL). On the other hand, this compound in DPPH displayed activity that was 8.4 times lower, and in the FRAP test, it did not reach IC_50_ values despite showing increasing concentrations until 2 mg/mL. Interestingly, the two Cu(II) complexes, **C1** and **C2**, showed moderate antioxidant properties in the DPPH assay (63.35—78.33 µg/mL). The weak antioxidant properties were displayed by ligand-bearing phthalazin-1(2*H*)-one moiety **L3** (see Table 8). Its IC_50_ values ranged from 198.66 µg/mL to 427.34 µg/mL. In terms of ligand **L1**, the obtained results may indicate some correlation between antiproliferative and antiradical activity. For the remaining compounds, especially complexes **C1** and **C2**, there is no such correlation. Due to this, their anticancer activity cannot be attributed to antioxidant properties.

### 2.8. In Silico Physicochemical, Pharmacokinetic, and Drug-Likeness Predictions for Ligands ***L1*** and ***L3*** and Cu(II) Complexes ***C1*** and ***C2***

The basic features of a drug molecule that determine its absorption and transport in the body include solubility, lipophilicity, charge, and size. According to Lipinski’s rules, undissociated substances with a molecular weight below 500 Da that are moderately lipophilic (logP in the range of 1–3) will be best absorbed.

**Figure 15:**
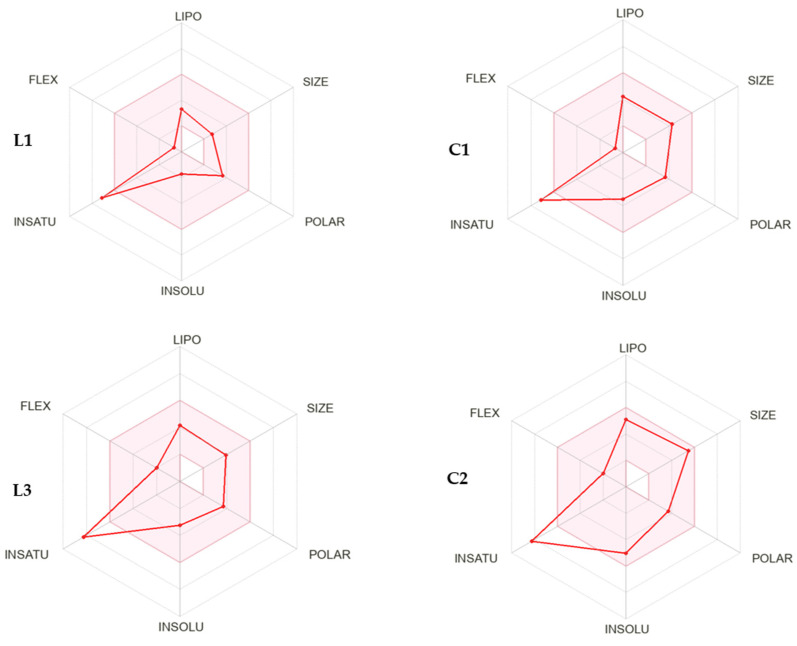
Oral bioavailability radar charts for the studied ligands (**L1** and **L3**) and Cu(II) complexes (**C1** and **C2**). The colored area shows the optimal range for each physicochemical property of oral bioavailability. LIPO—lipophilicity; SIZE—size; POLAR—polarity; INSOLU—solubility; INSATU—saturation; FLEX—flexibility.

**Figure 16:**
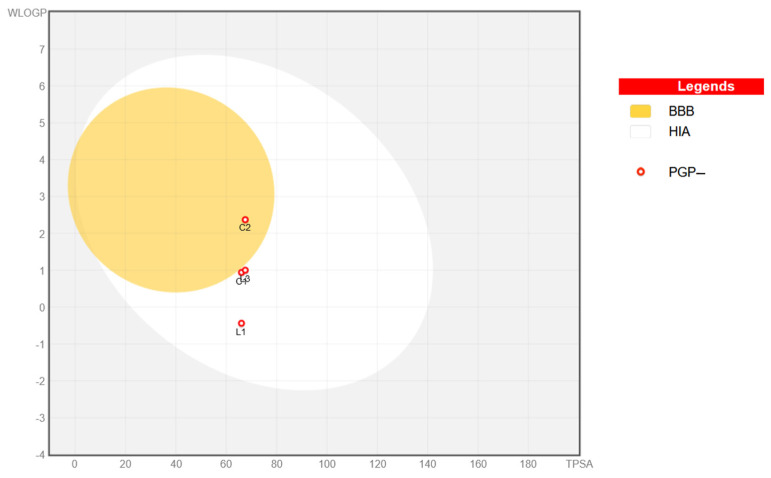
‘BOILED-egg’ plot for studied ligands, **L1** and **L3**, and their copper(II) complexes, **C1** and **C2**.

An estimation of the physicochemical characteristics, pharmacokinetic properties, and drug-likeliness of ligands **L1** and **L3** and the copper(II) complexes **C1** and **C2** was carried out using the freely available pre-ADMET web tool. The computed data are shown in Table 9 and Figure 15 and Figure 16 (see also Table S2 on page 12 in the Supplementary Materials for more details).

The Lipinski filter was applied to evaluate principal properties and confirm the drug-likeness of the molecules. Table 9 shows the calculated basic parameters of ligand **L3** and copper(II) complex **C2**. A consensus log value (ClogP) of the partition coefficient in octanol and water was calculated to assess the lipophilicity of the compounds synthesized in this study. The ClogP value must be within 5. As can be seen from Table 9, both ligand **L3** and copper(II) complex **C2** exhibit ClogP values within the range of 2.17–2.39.

In order to calculate the topological polar surface area (TPSA) [89,90,91], the carbon atoms, halogen atoms, and hydrogen atoms that are bonded to carbon atoms must first be subtracted from the molecular surface (i.e., nonpolar hydrogen atoms). Thus, it can be said that a TPSA is a surface containing polar hydrogen atoms along with other heteroatoms such as oxygen, nitrogen, and phosphorus. It is noteworthy that a heightened TPSA rate (>140 Å) is connected with low membrane permeability and poor blood–brain barrier access. Both the ligands and Cu(II) complexes possess favorable TPSA values of 59.60 Å^2^ and 67.56 Å^2^, respectively.

As indicated by Lipinski’s rule of 5, the lead structure must contain no more than five hydrogen bond donors (HBDs), whereas the number of hydrogen bond acceptors (HBAs) should be less than 10. The aforementioned standard was congregated since calculated ligands **L1** and **L3** and Cu(II) complexes **C1** and **C2** exhibit only three or four HBAs. Compounds are expected to possess high oral bioavailability, as required by the Veber rule. According to this, the number of rotatable bonds (ROTBs) must be equal to 10 and have a TPSA lower than 140 Å^2^. Otherwise, the entire number of hydrogen bond acceptors and donors (HBAs + HBDs) should be less than 12.

Cytochrome P-450 isoenzymes play a crucial role in drug biotransformation processes. The most significant hemoproteins are CYP1A2, CYP2C9, CYP2C19, CYP2D6, and CYP2E1, which are responsible for the transformation of nearly 90% of drugs. Cytochrome P-450 isoenzymes show structural diversity and interact differently with medicinal substances. Differences in xenobiotic affinity towards isozymes will significantly affect their elimination and concentration rates in bodily fluids. The metabolism of many endogenous substances and lipophilic xenobiotics—also classified as phase I biotransformation reactions—is based on oxidation reactions. As predicted in the pre-ADMET program, ligand **L3** inhibits CYP1A2 and CYP2C19 isoenzymes. On the other hand, complex compound **C2** inhibits only one isoenzyme, which may significantly affect its half-life.

According to Table 9, the tested molecules are characterized with suitable polarity with TPSA values ranging from 59.60 Å^2^ to 67.56 Å^2^. Optimal lipophilicity possess free ligand **L3** and its copper(II) complex **C2**, reaching ClogP values ranging from 2.17 to 2.39. Moreover, free ligands **L1** and **L3** are expected to be very soluble or soluble in water, whereas copper(II) chelates **C1** and **C2** display moderate solubility levels.

In line with the data shown in Table 9 and Figure 15 and Figure 16 (Bioavailable Radar Charts and ‘BOILED-Egg’ Plot), it may be concluded that synthesized ligands **L1** and **L3** and complexes **C1** and **C2** are predicted to have high gastrointestinal tract (GI) absorption. Interestingly, BBB permeant properties display only the copper(II) complex **C2** bearing a phthalazinone skeleton and a benzoyl group. Additionally, as shown in Table 9, both **L1** and **L3** as well as **C1** and **C2** meet all Lipinski’s ‘rule of five’ criteria as one of the key drug-likeness characteristics.

## 3. Materials and Methods

### 3.1. Experimental Part

#### 3.1.1. General Information

All solvents were purchased from a commercial supplier—Avantor Performance Materials Poland S.A., Gen. Sowińskiego 11, 44-121 Gliwice, Poland—and used without further purification. Benzoyl chloride (C_6_H_5_COCl), triethylamine (Et_3_N), anhydrous dichloroethane (DCE), and copper(II) chloride dihydrate (CuCl_2_ × 2H_2_O) were sourced from Sigma Chemical Co. (St. Louis, MO, USA). Synthesized compounds were purified using Preparative Thin-Layer Chromatography or Classical Column Chromatography. TLC was performed on Merck Silica Gel plates with fluorescence detection (under UV light, λ = 254 nm). Melting point (m.p.) measurements were performed on a B-545 apparatus (BÜCHI, Flawil, Switzerland). Infrared spectra of synthesized compounds were collected on a Nicolet 380 FT-IR spectrophotometer (Thermo, Waltham, MA, USA). Elemental analyses of C, H, and N of all compounds were within ± 0.4% of theoretical values. Nuclear magnetic spectra of proton and carbon atoms (^1^H and ^13^C NMR) were recorded using a Bruker (Billerica, MA, USA) Advance III HD 400 MHz spectrometer in deuterated dimethyl sulfoxide (DMSO-*d_6_*). Solvent signals were an internal standard. Chemical shifts (*δ*) are reported in *ppm* relative to residual solvent signals. Coupling constants are shown in hertz (*J*, Hz). Mass spectra of ligands **L1**, **L2**, and **L3** were measured on a Shimadzu (Kyoto, Japan) LCMS 2010 spectrometer (positive electrospray ionization). Stability of Cu(II) complexes **C1** and **C2** was assessed with a Perkin-Elmer Lambda UV/VIS spectrophotometer (Evolution 300 UV–Vis, Thermo Electron Scientific Instruments LLC, Madison, WI, USA connected to a personal computer running the VISION pro software vision version 4.4.1., math version 24.00). Diffraction experiments were carried out at a temperature in the range of 20–22 °C with an Oxford Diffraction Xcalibur E diffractometer (Agilent Technologies Inc., Santa Clara, CA, USA) using Mo Kα radiation for **L3** and with an Oxford Diffraction SuperNova diffractometer using Cu Kα radiation for **C2**. Diffraction data were processed with CrysAlisPro software [92]. The structures were solved with the program SHELXT [93] and refined by the full-matrix least-squares method on *F*^2^ with SHELXL-2019 [94] within Olex2 software [95]. Hydrogen atoms were placed in calculated positions and refined as they rode on their carriers. Crystallographic data and structure refinement details are shown in Table 1. CCDC 2412635-2412636 contain supplementary crystallographic data. These data can be obtained free of charge via http://www.ccdc.cam.ac.uk/conts/retrieving.html, accessed on 1 December 2024 (or from the CCDC, 12 Union Road, Cambridge CB2 1EZ, UK; Fax: +44-1223-336033; E-mail: deposit@ccdc.cam.ac.uk). The physicochemical, pharmacokinetic, and drug-likeness properties of **L1**, **L3**, **C1**, and **C2** were predicted using the SwissADME program available online, SwissADME Swiss Drug Design, at www.swissadme.ch (accessed on 1 December 2024).

#### 3.1.2. Preparation of Free Ligands: *N*-(2-(1-Benzoyl-4,5-dihydro-1*H*-imidazol-2-yl)phthalazin-1(2*H*)-ylidene)benzamide (**L2**) and 2-(1-Benzoyl-4,5-dihydro-1*H*-imidazol-2-yl)phthalazin-1(2*H*)-one (**L3**)

##### First Step—Synthesis of Ligand **L2** and Its Purification

To the mixture of **L1** (0.213 g, 1 mmol) and Et_3_N (0.304 g, 0.42 mL, d = 0.726 g/mL, 3 mmol) in anhydrous DCE (5 mL), benzoyl chloride (0.281 g, 0.23 mL, d = 1.211 g/mL, 2 mmol) was added dropwise. The reaction mixture was heated at 80–90 °C for 1 h, monitored by TLC (eluent EtOAc), and evaporated in vacuum. Then, the semi-solid residue was treated with a Na_2_CO_3_ solution at a concentration of 10–15 mL for 30 min and extracted with chloroform (3 × 15 mL). Organic layers were combined, dried over anhydrous MgSO_4_, filtered off, and evaporated under a reduced pressure until dried. To the crude **L2**, 5 mL of methanol was added. Precipitate was filtered off and purified using thin-layer preparative chromatography (eluent: mixtures of DCM and EtOAc in different concentrations). Isolated yield: 0.24 g (57%, white solid), m.p. 178–179 °C; IR (KBr) *v* [cm^−1^]: 3057, 3038, 3009, 2974, 2923, 2910, 2872, 1665, 1638, 1577, 1449, 1390, 1357, 1311, 1294, 1277, 1180, 1159, 1074, 1052, 1038, 1019, 994, 898, 710, 676; ^1^H-NMR (DMSO-*d*_6_, 400 MHz) *δ* [ppm]: 3.73 (t, 2H, CH_2_), 4.05 (t, 2H, CH_2_), 7.08 (m, 3H, 3xCH-Ar), 7.49–7.53 (m, 2H, 2xCH-Ar), 7.55–7.57 (m, 2H, 2xCH-Ar), 7.59–7.61 (m, 1H, CH-Ar), 7.80–7.83 (m, 1H, CH-Ar), 7.83–7.85 (m, 1H, CH-Ar), 7.88–7.90 (m, 1H, CH-Ar), 8.04–8.07 (m, 2H, 2xCH-Ar), 8.20 (d, *J* = 7.9, 1H, CH-Ar), 8.23 (s, 1H, CH-Ar); ^13^C-NMR (DMSO-*d*_6_, 100 MHz) *δ* [ppm]: 48.78, 49.78, 126.14, 127.03, 127.15, 127.86, 127.90 (two overlapping signals), 128.04 (two overlapping signals), 128.90 (two overlapping signals), 129.47 (two overlapping signals), 130.83, 132.96, 133.56, 134.68, 135.27, 135.78, 141.12, 144.72, 150.99, 167.37, 174.37; MS (ESI, MeOH:MeCN containing 0.1% AcOH, 1:1, *v*/*v*): *m*/*z* = 422 [M + H]^+^, *m*/*z* = 444 [M + Na]^+^ and *m*/*z* = 485 [M + Na + MeCN]^+^. Anal. calcd for C_25_H_19_N_5_O_2_ (421.45): C, 71.25; H, 4.54; N, 16.62. Found: C, 70.85; H, 4.48; N, 16.69.

##### Second Step—Synthesis of Ligand **L3**

The crude sample of ligand **L2** (0.427 g) was dissolved in chloroform (3–5 mL) and purified using classical column chromatography (eluent: EtOAc). Yield: 0.15 g (31.5%), m.p.: 164–165 °C; IR (KBr) *v* [cm^−1^]: 3065, 3050, 2936, 2876, 1678, 1635, 1600, 1452, 1379, 1359, 1320, 1279, 1250, 1170, 722, 714; ^1^H-NMR (DMSO-*d_6_*, 400 MHz) *δ* [ppm]: 3.97–4.02 (m, 2H, CH_2_), 4.15–4.19 (m, 2H, CH_2_), 6.96–7.06 (m, 3H, 3xCH-Ar), 7.28–7.31 (m, 2H, 2xCH-Ar), 7.78–7.83 (m, 2H, 2xCH-Ar), 7.89–7.92 (m, 1H, CH-Ar), 8.00 (d, *J* = 7.8 Hz, 1H, CH-Ar), 8.40 (s, 1H, CH-Ar); ^13^C-NMR (DMSO-*d_6_*, 100 MHz) *δ* [ppm]: 48.93, 50.85, 126.19, 126.47, 127.49, 127.77, 127.84 (three overlapping signals), 129.45, 130.63, 132.99, 134.78, 135.02, 140.50, 151.03, 158.46, 167.46; MS (ESI, MeOH:MeCN containing 0.1% AcOH, 1:1, *v*/*v*): *m/z* = 319 [M + H]^+^, *m/z* = 341 [M + Na]^+^ and *m/z* = 382 [M + Na + MeCN]^+^. Anal. calcd for C_18_H_14_N_4_O_2_ (318.33): C, 67.91; H, 4.43; N, 17.60. Found: C, 67.70; H, 4.50; N, 17.47.

#### 3.1.3. Synthesis of Copper(II) Complexes **C1** and **C2**

##### Synthesis of Dichloro[2-(4,5-dihydro-1*H*-imidazol-2-yl)phthalazin-1(2*H*)-imine]copper(II) (**C1**)

Copper(II) chloride dihydrate CuCl_2_ × 2H_2_O (0.341 g, 2 mmol) dissolved in 1 mL of 99.5% dimethylformamide (DMF) was added dropwise to a stirred solution of ligand **L1** (0.2132 g, 1 mmol) in 5 mL of 99.5% DMF at room temperature (20–22 °C). Then, after 1 h of stirring, the green precipitate of copper(II) complex **C1** was filtered off and washed with a small amount of DMF. Yield: 0.125 g (36%), m.p.: 284–286 °C, IR (KBr) *v* [cm^−1^]: 3401, 3275, 3107, 3062, 3030, 2974, 2898, 1646, 1601, 1502, 1468, 1316, 1282, 1219, 1143, 969, 905, 772, 731, 698 587. Anal. calcd for C_11_H_11_Cl_2_CuN_5_ (347.69): C, 38.00; H, 3.19; N, 20.14. Found: C, 37.81; H, 3.29; N, 19.98.

##### Synthesis of Dichloro[2-(1-benzoyl-4,5-dihydro-1*H*-imidazol-2-yl)phthalazin-1(2*H*)-one]copper(II) (**C2**)

To a hot solution (80 °C) of ligand **L3** (0.1 g, 0.314 mmol) in 99.5% DMF (3–4 mL), copper(II) chloride dihydrate CuCl_2_ × 2H_2_O (0.107 g, 0.628 mmol) dissolved in 1 mL of DMF was added. Upon slow evaporation of the solvent over 1–2 days at a temperature range of 20–22 °C, green crystals of copper(II) complex **C2** were formed. Crystals were filtered off, washed with DMF (approx. 0.5 mL), and dried. Yield: 0.06 g (42%), m.p.: 285 °C (decomposition), IR (KBr) *v* [cm^−1^]: 3060, 2923, 1710, 1689, 1628, 1604, 1592, 1574, 1428, 1346, 1294, 1280, 1262, 995, 714, 700. Anal. calcd for C_18_H_14_Cl_2_CuN_4_O_2_ (452.78): C, 47.75; H, 3.12; N, 12.37. Found: C, 47.53; H, 3.02; N, 12.58.

### 3.2. Stability Studies of Dichloro[2-(4,5-dihydro-1H-imidazol-2-yl)phthalazin-1(2H)-imine]copper(II) (***C1***) and Dichloro[2-(1-benzoyl-4,5-dihydro-1H-imidazol-2-yl)phthalazin-1(2H)-one]copper(II) (***C2***)

An amount of 10 µL of 20 mM solutions of copper(II) complex (**C1** or **C2**) in dimethyl sulfoxide (DMSO) was added to 5.0 mL of phosphate-buffered saline (PBS, pH 7.4). Then, the resulting 40 µM solutions of Cu(II) complexes were transferred to a 1.0 cm quartz cuvette, and UV-Vis spectra (λ = 200–600 nm) were recorded at 120 min intervals over 12 h.

### 3.3. Biology

#### 3.3.1. Evaluation of In Vitro Cytotoxicity

##### Cell Culture

Cytotoxicity of synthesized complexes was tested on two human breast cancer cell lines (MDA MB 231 and MCF-7), human cervical cancer cells (HeLa), and non-cancer human skin fibroblast cells (HDF) obtained from the American Type Culture Collection (ATCC; Manassas, VA, USA). The cells were cultured in PAN Biotech DMEM supplemented with 10% fetal bovine serum (FBS), 6 μg/mL of penicillin-G, and 10 μg/mL of streptomycin.

##### The MTT Assay

To check the viability of the cell lines, an MTT assay was used. MDA MB 231, MCF-7, and HDF cells were seeded in triplicate in 96-well plates at a density of 10,000 cells/well, and HeLa cells were seeded at a density of 5000 cells/well in a cell culture medium. The cells were treated with synthesized complexes for 48 h at concentrations in the range of 1–200 μM. Cisplatin was used for comparison purposes. Absorbance was recorded at 570/660 nm (ASYS Hitech GmbH microplate reader, Biogenet, Parkingowa 1, 05-420 Józefów, Poland). The compound blank was used for each concentration to correct the absorbance values for the background. The results were calculated as a percentage of the control values (unexposed cells set up as 100%).

##### Statistical Analysis

Statistical analysis was carried out using GraphPad Prism (available on line: https://www.graphpad.com/, accessed on 1 December 2024). The experiments were carried out in triplicate, and the results are presented as the mean ± SD. Statistical analysis was conducted by using a one-way analysis of variance, followed by Tukey’s or Dunnett’s post hoc test.

#### 3.3.2. Determination of Minimum Inhibitory Concentration (MIC) and Minimum Bactericidal/Fungicidal Concentration (MBC/MFC)

The MIC and MBC of the tested compounds were determined by the microbroth dilution method according to the European Committee on Antimicrobial Susceptibility Testing (EUCAST) [74,75] against reference strains of the following microorganisms: *Staphylococcus aureus* strain ATCC 6538, *Escherichia coli* strain ATCC 8739, and *Candida albicans* strain ATCC 10231. Dry test samples were dissolved in DMSO (dimethyl sulfoxide) to give a stock concentration of 15 mg/mL, and the studies were conducted in the concentration range of 300–3.1 µg/mL using 96-well plates. Control samples with Ciprofloxacin for bacteria and Amphotericin B for yeast were also used. The cultures of bacteria were prepared by transferring cells from the stock cultures to tubes with MH (Mueller–Hinton) broth and incubated with agitation for 18 ± 2 h at 35 ± 1 °C. To culture *C. albicans* strain ATCC 10231, RPMI 1640 broth + MOPS buffer and incubation at the same conditions were used. The cultures were diluted with the appropriate broth to achieve an optical density corresponding to 5 × 10^5^ (bacteria) or 2 × 10^5^ (yeast) colony-forming units per mL (cfu/mL). MH or RPMI 1640 broth + MOPS buffer in volumes of 96 µL and 4 µL of the proper concentration of the tested compounds and next microbial suspension (100 µL) were added to each well. Then, 96-well plates were incubated at 35 ± 1 °C for 18 ± 2 h, and microbial growth was assessed. The MIC was taken as the lowest concentration of the agent that completely inhibits visible growth (bacteria) [74] or complete (>90%) inhibition for Amphotericin B but 50% growth inhibition for other compounds (spectrophotometric) [75]. Next, 100 µL of the suspension from each well without growth was inoculated on appropriate agar plates to control microbial viability. After incubation for 18 ± 2 h at 35 ± 1 °C, the CFU was determined. The MBC/MFC was defined as the minimal concentration of tested compounds required to kill 99.9% the organisms [96,97].

#### 3.3.3. Antioxidant Studies

##### Materials

2,2-Diphenyl-1-picrylhydrazyl—DPPH; 2,2′-azino-bis(3-ethylbenzothiazoline-6-sulfonic acid diammonium salt—ABTS; potassium persulfate—K_2_S_2_O_8_; 2,4,6-tris(2-pyridyl)-*s*-triazine—TPTZ; dimethyl sulfoxide—DMSO; and L-ascorbic acid were sourced from Sigma Chemical Co. Hydrochloric acid—HCl; ferric chloride hexahydrate—FeCl_3_ × 6H_2_O; acetic acid—AcOH; chloroform; Polyoxyethylene Sorbitan Monopalmitate—Tween-40; and methanol of HPLC grade were sourced from Avantor Performance Materials Poland S.A.

##### DPPH Radical Scavenging Assay

A radical scavenging assay with 2,2-diphenyl-1-picrylhydrazyl (DPPH) was performed by mixing 100 μL of the tested compounds dissolved in DMSO at different concentrations with 100 μL of 0.06 mM DPPH methanolic solution and incubating at room temperature in the dark for 30 min. L-ascorbic acid was used as a positive control [98,99]. A 96-well microplate reader (Epoch, BioTek System, Winooski, VT, USA) was used to measure changes in absorbance at a wavelength of λ = 517 nm. In the control solution, DPPH was combined with DMSO. The following equation was used to calculate DPPH inhibition:

DPPH Inhibition (%) = [(A_control_ − A_sample_)/A_control_] × 100%

In the experiments, the radical scavenging properties of the samples were expressed as IC_50_ values—the concentration that caused a 50% reduction in the non-reduced form of the DPPH radical.

##### ABTS Radical Scavenging Assay

A radical scavenging assay with 2,2′-azino-bis(3-ethylbenzothiazoline-6-sulfonic acid diammonium salt—ABTS—was performed by mixing 30 μL of tested compounds dissolved in DMSO at different concentrations with 170 μL of ABTS (2 mM ABTS and 3.5 mM K_2_S_2_O_8_) and water to a final volume of 300 μL. L-ascorbic acid was used as a positive control [98,99]. In the control solution, ABTS was combined with DMSO. After 10 min of incubation in the dark at a temperature of 30 °C, the change in absorbance was observed at a wavelength of λ = 750 nm using a 96-well microplate reader (Epoch, BioTek System). The following equation was used to calculate ABTS inhibition:

ABTS Inhibition (%) = [(A_control_ − A_sample_)/A_control_] × 100%

In the experiments, the radical scavenging properties of the samples were expressed as IC_50_ values—the concentration that caused a 50% reduction in the non-reduced form of the ABTS radical.

##### FRAP Test

A FRAP test was performed to determine the reducing ability of tested compounds based on Fe^3+^ reduction to Fe^2+^ [100]. Serial dilutions of the tested compounds (30 μL) and standard substance were placed in a 96-well plate and mixed with 170 μL of the freshly prepared reaction mixture containing 0.3 M acetate buffer, 10 mM TPTZ in 40 mM HCl, and 20 mM FeCl_3_ × 6H_2_O at a ratio of 10:1:1 (*v*/*v*/*v*). An absorbance measurement at a wavelength of λ = 593 nm was taken after the plate was incubated for 20 min at room temperature. In order to calculate the percentage of reduced iron ions, the calibration curve for ascorbic acid (1–1000 µg/mL) was plotted. We conducted three independent analyses, each with three replicates. The IC_50_ value—the concentration of the analyzed extract or standard substance (L-ascorbic acid) that reduces Fe^3+^ ions by 50%—was calculated using GraFit v.7.0 Erithacus Software.

## 4. Conclusions

In this work, two copper(II) complexes, **C1** and **C2**, derived from phthalazine and imidazoline containing ligands **L1** and **L3** were synthesized. The structures of the ligands and Cu(II) complexes were confirmed by IR spectroscopy and elemental analysis. Moreover, for ligand **L3** and copper(II) complex **C2**, single-crystal X-ray diffraction studies were conducted. As evidenced by the crystal structure of **C2**, the binding of the copper ion strongly influences ligand **L3**’s conformation and the carbonyl–carbonyl interaction. In copper(II) complex **C2**, the coordination environment of the metal ion can be described as a distorted square planar. Additional contact between the inversion center of related molecules assembles the monomeric molecules into a dinuclear complex with a {Cu_2_(μ_2_-Cl)_2_} core that forms a distorted square pyramid.

The stability of Cu complexes **C1** and **C2** in an aqueous buffer solution (pH 7.4) was investigated using UV-Vis spectroscopy, and the recorded spectra showed no noticeable changes over 12 h of incubation. A density functional (B3LYP) approach was used to evaluate the possible conformations and electronic structure of ligand **L3**. Theoretical calculations revealed that the equilibrium lies quantitatively on the side of the *E*-conformation of ligand **L3** with a torsion angle of 180°.

The in vitro anticancer potency of free ligands **L1** and **L3** and their corresponding copper(II) complexes **C1** and **C2** was evaluated against human cancers, namely the cervix epithelial cell line (HeLa), breast epithelial-like adenocarcinoma (MCF-7), and breast epithelial cancer cell line (MDA-MB-231), by use of an MTT assay after 48 h of incubation. The most potent compound was copper(II) complex **C2** bearing a phthalazin-1(2*H*)-one scaffold. According to in vitro tests, **C2** reduced the viability of HeLa, MCF-7, and MDA-MB-231 cells by about 57.5–81.2%. Copper(II) complex **C2** at a concentration of 200 μg/mL demonstrated comparable anticancer potency against triple-negative breast cancer (MDA-MB-231) compared to Cisplatin (cancer cells with viability of 23.6% vs. 22.6%).

The antimicrobial activity of ligands **L1** and **L3** and their copper(II) complexes **C1** and **C2** was evaluated using reference strains of the following bacteria and yeasts: *Staphylococcus aureus*, *Escherichia coli*, and *Candida albicans*. It was evident that all compounds were devoid of antibacterial or antifungal activity. This feature seems to be beneficial in terms of their potential uses as anticancer agents in the future.

The antioxidant properties of ligands and their copper(II) complexes were screened with three methods: ABTS, DPPH, and FRAP. In vitro assays revealed that a moderate antiradical effect was observed for free ligand **L1** containing phthalazin-1(2*H*)-imine in the ABTS radical scavenging assay (IC_50_ = 23.63 µg/mL) compared with L-ascorbic acid. **L1** also displayed a moderate anticancer effect.

Finally, the ADME technique was used to determine the physicochemical, pharmacokinetic, and drug-likeness properties of ligands **L1** and **L3** and their corresponding copper(II) complexes **C1** and **C2**. Thus, a preliminary biological evaluation and in silico drug-likeness prediction can be useful for developing new anticancer agents.

In terms of the molecular targets of **C1** and **C2** to date, a variety of mechanisms have been observed to benefit copper coordination compounds in anticancer treatment. A crucial aspect of the anticancer activity of copper complexes may be the inhibition of topoisomerases I and II (Top1 and Top2). Many Top inhibitors are represented by mononuclear copper(II) complexes that coordinate with different organic ligands. By forming ternary complexes with an enzyme and DNA, copper complexes promote DNA damage. Consequently, this disrupts DNA topology, leading to cell cycle arrest and death. The inhibition of Top2 might be a good therapeutic option for breast cancer, including triple-negative breast cancer [101,102]. Additionally, as a result of modulating apoptotic regulatory proteins in breast cancer cells, copper(II)-based agents may cause apoptotic cell death [103]. However, at this stage of work, it is too early to speculate on the mechanism of action of the tested copper(II) complexes. Thus, in the next step, we will assess a broad spectrum of phthalazine-based derivatives to find more active compounds for further studies on their anticancer mechanism.

## Figures and Tables

**Table 1:** Crystal data and details of structure refinement.

Compound Reference	L3	C2
Chemical formula	C_18_H_14_N_4_O_2_	C_18_H_14_N_4_O_2_Cl_2_Cu
Formula mass	318.33	452.77
Crystal system	triclinic	triclinic
*a*/Å	8.3830(3)	7.38342(18)
*b/*Å	8.7581(2)	10.0507(3)
*c/*Å	10.8035(3)	12.8296(3)
α/°	102.174(3)	91.220(2)
β°	102.877(3)	91.9414(18)
ɣ°	91.121(2)	110.026(2)
Unit cell volume/Å^3^	754.00(4)	893.44(4)
Space group	*P*-1	*P*-1
Z	2	2
Radiation type	Mo *K*α	Cu *K*α
Absorption coefficient, μ/mm^−1^	0.095	4.673
No. of refl. measured, 2Θ_max_/°	20,402, 52.7	22,327, 153.1
No. of independent reflections	3074	3714
*R*_int_	0.0182	0.0276
Final *R*_1_ values (*I* > 2σ(I))	0.0402	0.0266
Final wR(F^2^) values (I > 2σ(I))	0.0906	0.0734
Final *R*_1_ values (all data)	0.0453	0.0285
Final wR(F^2^) values (all data)	0.1008	0.0751
Goodness of fit on F^2^	1.037	1.059

**Table 2:** Geometry of C=O···C=O interaction in **L3** and **C2**.

Compound Reference	L3	C2
d_O···C_ (Å)	2.9547(17)	2.655(2)
Θ_O···C=O_ (°)	114.31(10)	113.42(13)
T_C=O···C=O_ (°)	169.83(12)	169.82(16)

**Table 3:** Selected geometric parameters of **L3** and **C2** molecules.

Compound Reference	L3	C2
Bond lengths (Å)		
C1-O16	1.2057(16)	1.209(2)
C1-N2	1.3846(17)	1.417(2)
N2-N3	1.3966(16)	1.4006(18)
N2-C11	1.4142(17)	1.397(2)
C11-N15	1.2612(18)	1.283(2)
C11-N12	1.3963(17)	1.367(2)
N12-C17	1.3788(18)	1.408(2)
C17-O18	1.2146(16)	1.207(2)
Cu1-Cl1		2.2262(5)
Cu1-Cl2		2.2420(5)
Cu1-N3		2.0844(13)
Cu1-N15		1.9634(15)
Bond angles (°)		
C4 N3 N2	115.31(11)	118.03(13)
C11 N15 C14	105.60(12)	107.55(14)
C11 N2 C1	119.15(11)	123.44(13)
N12 C11 N2	120.63(11)	125.00(14)
C11 N12 C17	131.66(11)	133.28(14)
Torsion angles (°)		
N3 N2 C11 N15	55.60(18)	14.3(2)
C11 N12 C17 C19	36.1(2)	30.4(3)
N12 C17 C19 C24	37.58(19)	24.7(2)

**Table 4:** The effect of ligands **L1** and **L3** and complexes **C1** and **C2** in different concentrations [μg/mL] on the viability (%) of cervical cancer cells in the HeLa line after 48 h of incubation. An MTT assay was used for measuring cell viability. *Cisplatin was used as a positive control. The values shown are the means from three independent experiments.

	HeLa Cell Line					
Conc. [μg/mL]	1	10	25	50	100	200
Compound	Mean % of Control					
**L1**	100	94.5	91.8	79.3	58.1	34.1
**C1**	97.3	97.1	97.4	93.9	91.0	56.4
**L3**	96.5	92.1	92.4	94.8	94.1	86.4
**C2**	99.9	97.5	93.3	95.2	77.7	18.8
Cisplatin*	93.1	90.3	80.6	72.3	5.0	2.5

**Table 5:** The effect of ligands **L1** and **L3** and complexes **C1** and **C2** in different concentrations [μg/mL] on the viability (%) of human breast epithelial-like adenocarcinoma, line MCF-7, after 48 h of incubation. An MTT assay was used for measuring cell viability. *Cisplatin was used as a positive control. The values shown are the means from three independent experiments.

	MCF-7 Cell Line					
Conc. [μg/mL]	1	10	25	50	100	200
Compound	Mean % of Control					
**L1**	97.3	97.2	93.7	86.4	62.1	45.7
**C1**	100	100	100	97.9	86.2	49.2
**L3**	95.9	100	97.2	99.4	96.5	95.6
**C2**	100	100	96.4	92.5	73.7	42.5
Cisplatin*	100	96.4	81.9	64.1	18.8	6.5

**Table 6:** The effect of ligands **L1** and **L3** and complexes **C1** and **C2** in different concentrations [μg/mL] on the viability (%) of triple-negative breast cancer cells, line MDA-MB-231, after 48 h of incubation. An MTT assay was used for measuring cell viability. *Cisplatin was used as a positive control. The values shown are the means from three independent experiments.

	MDA-MB-231 Cell Line					
Conc. [μg/mL]	1	10	25	50	100	200
Compound	Mean % of Control					
**L1**	99.3	97.8	95.0	81.4	52.7	35.2
**C1**	90.5	88.8	89.6	81.7	80.5	18.7
**L3**	97.2	97.6	96.6	94.9	96.2	96.7
**C2**	96.2	97.5	93.5	88.9	70.5	23.6
Cisplatin*	98.2	87.7	82.4	57.6	31.9	22.6

**Table 7:** The effect of ligand **L1** and complexes **C1** and **C2** in different concentrations [μg/mL] on the viability (%) of skin fibroblasts, HDFa line, after 48 h of incubation. An MTT assay was used for measuring cell viability. *Cisplatin was used as a positive control. The values shown are the means from three independent experiments.

	HDFa Cell Line		
Conc. [μg/mL]	50	100	200
Compound	Mean % of Control		
**L1**	85.6	81.3	84.8
**C1**	-	86.9	75.6
**C2**	-	78.8	68.1
Cisplatin*	76.8	26.8	4.1

**Table 8:** Antioxidant properties of ligands **L1** and **L3** and Cu(II) complexes **C1** and **C2** expressed as IC_50_ values [µg/mL] (±SD, standard deviation).

Compound	ABTS	DPPH	FRAP
**L1**	23.63 ± 0.28	197.89 ± 1.79	NR *
**L3**	198.66 ± 1.33	427.34 ± 2.25	214.28 ± 0.2
**C1**	253.92 ± 2.15	63.35 ± 0.25	156.01 ± 0.35
**C2**	400.37 ± 0.87	78.33 ± 1.13	158.32 ± 0.21
Ascorbic acid	15.68 ± 0.8	12.75 ± 0.55	10.17 ± 0.51

* NR—the IC_50_ value was not reached.

**Table 9:** Predicted physicochemical, pharmacokinetic, and drug-likeness properties of synthesized compounds **L1**, **L3**, **C1**, and **C2**.

	Physicochemical Properties					Lipophilicity	Water Sol.	Pharmacokinetics				Drug-Likeness
	Mol. Wt.	ROTB (n)	HBA (n)	HBD (n)	TPSA	CLogP	Solubility Class	GI abs.	BBB perm.	CYP1A2 *	CYP2C19 *	Lipinski Filter
Rule	<500	<10	<10	<5	-	<5	-	-	-	-	-	-
**L1**	213.24	1	3	2	66.06	1.07	soluble (v)	high	No	Yes	No	Yes(0)
**L3**	318.33	3	4	0	59.60	2.17	soluble	high	No	Yes	Yes	Yes(0)
**C1**	347.69	1	3	2	66.05	1.41	soluble	high	No	Yes	No	Yes(0)
**C2**	452.78	3	4	0	67.56	2.39	soluble (m)	high	Yes	Yes	No	Yes(0)

Mol. Wt.—molecular weight (g/mol); (n)—number; ROTB—rotatable bonds; HBA—hydrogen bond acceptor; HBD—hydrogen bond donor; TPSA—topological polar surface area (Å^2^); CLogP—consensus log of partition coefficient; m—moderate; v—very; Lipinski filter with number of violations in brackets; GI—gastrointestinal absorption; BBB—blood–brain barrier permeant; CYP1A2 * and CYP2C19 * inhibitor.

## Data Availability

All data are contained within the article and Supplementary Materials.

## Supplementary Materials

The following supporting information can be downloaded at https://www.mdpi.com/article/10.3390/ph18030375/s1, Figure S1: IR spectrum of 2-(4,5-dihydro-1*H*-imidazol-2-yl)phthalazin-1(2*H*)-imine (ligand **L1**); Figure S2: IR spectrum of *N*-(2-(1-benzoyl-4,5-dihydro-1*H*-imidazol-2-yl)phthalazin-1(2*H*)-ylidene)benzamide (ligand **L2**); Figure S3: ^1^H-NMR spectrum of *N*-(2-(1-benzoyl-4,5-dihydro-1*H*-imidazol-2-yl)phthalazin-1(2*H*)-ylidene)benzamide (ligand **L2**) registered in DMSO-*d*_6_ (400 MHz); Figure S4: ^13^C-NMR spectrum of *N*-(2-(1-benzoyl-4,5-dihydro-1*H*-imidazol-2-yl)phthalazin-1(2*H*)-ylidene)benzamide (ligand **L2**) registered in DMSO-*d*_6_ (100 MHz); Figure S5: Mass spectrum of *N*-(2-(1-benzoyl-4,5-dihydro-1*H*-imidazol-2-yl)phthalazin-1(2*H*)-ylidene)benzamide (ligand **L2**); Figure S6: IR spectrum of 2-(1-benzoyl-4,5-dihydro-1*H*-imidazol-2-yl)phthalazin-1(2*H*)-one (ligand **L3**); Figure S7: ^1^H-NMR spectrum of 2-(1-benzoyl-4,5-dihydro-1*H*-imidazol-2-yl)phthalazin-1(2*H*)-one (ligand **L3**) registered in DMSO-*d*_6_ (400 MHz); Figure S8: ^13^C-NMR spectrum of 2-(1-benzoyl-4,5-dihydro-1*H*-imidazol-2-yl)phthalazin-1(2*H*)-one (ligand **L3**) registered in DMSO-*d*_6_ (100 MHz); Figure S9: Mass spectrum of 2-(1-benzoyl-4,5-dihydro-1*H*-imidazol-2-yl)phthalazin-1(2*H*)-one (ligand **L3**); Figure S10: IR spectrum of dichloro[2-(4,5-dihydro-1*H*-imidazol-2-yl)phthalazin-1(2*H*)-imine]copper(II) (complex **C1**); Figure S11: IR spectrum of dichloro[2-(1-benzoyl-4,5-dihydro-1*H*-imidazol-2-yl)phthalazin-1(2*H*)-one]copper(II) (complex **C2**); Figure S12: CheckCIF/PLATON report of 2-(1-benzoyl-4,5-dihydro-1*H*-imidazol-2-yl)phthalazin-1(2*H*)-one (ligand **L3**); Figure S13: CheckCIF/PLATON report of dichloro[2-(1-benzoyl-4,5-dihydro-1*H*-imidazol-2-yl)phthalazin-1(2*H*)-one]copper(II) (complex **C2**); Table S1: Minimum inhibitory concentration (MIC) and minimum bactericidal concentration (MBC) [µg/mL] with the standard deviation (±SD) of the free ligands **L1**, **L3** and their copper(II) complexes **C1**, **C2** on reference strains of bacteria and yeasts; Table S2: Predicted physicochemical, pharmacokinetic and drug-likeness properties of ligands **L1**, **L3** and their copper(II) complexes **C1**, **C2**.
