# Supplementary material for: Synthesis, Structure, and Stability of Copper(II) Complexes Containing Imidazoline-Phthalazine Ligands with Potential Anticancer Activity
Source: Pharmaceuticals (Basel). 2025 Mar 6;18(3):375. doi: 10.3390/ph18030375 (PMC11946467; doi:10.3390/ph18030375)
Supplement: Supplementary file 1 [file pharmaceuticals-18-00375-s001.zip › pharmaceuticals-3479488-supplementary.pdf]

Supplementary Materials

Synthesis, Structure, and Stability of Copper(II) Complexes Containing Imidazoline-Phthalazine Ligands with Potential Anticancer Activity

Łukasz Balewski [1,*], Iwona Inkielewicz-Stępniak [2], Maria Gdaniec [3], Katarzyna Turecka [4], Anna Hering [5], Anna Ordyszewska [6], and Anita Kornicka [1]

1 Department of Chemical Technology of Drugs, Faculty of Pharmacy, Medical University of Gdansk, Gen. J. Hallera 107, 80-416 Gdańsk, Poland
2 Department of Pharmaceutical Pathophysiology, Faculty of Pharmacy, Medical University of Gdansk, Gen. Dębinki 7, 80-211, Gdańsk, Poland
3 Faculty of Chemistry, Adam Mickiewicz University, Uniwersytetu Poznańskiego 8, 61-614 Poznań, Poland
4 Department of Pharmaceutical Microbiology, Faculty of Pharmacy, Medical University of Gdansk, Gen. J. Hallera 107, 80-416 Gdańsk, Poland
5 Department of Biology and Pharmaceutical Botany, Faculty of Pharmacy, Medical University of Gdansk, Gen. J. Hallera 107, 80-416 Gdańsk, Poland
6 Department of Inorganic Chemistry, Faculty of Chemistry and Advanced Materials Centers, Gdańsk University of Technology, Narutowicza 11/12, 80-233 Gdansk, Poland

* Correspondence: lukasz.balewski@gumed.edu.pl

Table of contents:

1. Spectra of ligands L1, L2, L3, and copper(II) complexes C1 and C2

Figure S1. IR spectrum* of 2-(4,5-dihydro-1*H*-imidazol-2-yl)phthalazin-1(2*H*)-imine (ligand **L1**) **3**

Figure S2. IR spectrum of *N*-(2-(1-benzoyl-4,5-dihydro-1*H*-imidazol-2-yl)phthalazin-1(2*H*)-ylidene)benzamide (ligand **L2**) **3**

Figure S3. ^{1}H-NMR spectrum of *N*-(2-(1-benzoyl-4,5-dihydro-1*H*-imidazol-2-yl)phthalazin-1(2*H*)-ylidene)benzamide (ligand **L2**) registered in DMSO-d_6 (400 MHz) **4**

Figure S4. ^{13}C-NMR spectrum of *N*-(2-(1-benzoyl-4,5-dihydro-1*H*-imidazol-2-yl)phthalazin-1(2*H*)-ylidene)benzamide (ligand **L2**) registered in DMSO-d_6 (100 MHz) **4**

Figure S5. Mass spectrum of *N*-(2-(1-benzoyl-4,5-dihydro-1*H*-imidazol-2-yl)phthalazin-1(2*H*)-ylidene)benzamide (ligand **L2**) **5**

Figure S6. IR spectrum of 2-(1-benzoyl-4,5-dihydro-1*H*-imidazol-2-yl)phthalazin-1(2*H*)-one (ligand **L3**) **6**

Figure S7. ^{1}H-NMR spectrum of 2-(1-benzoyl-4,5-dihydro-1*H*-imidazol-2-yl)phthalazin-1(2*H*)-one (ligand **L3**) registered in DMSO-d_6 (400 MHz) **6**

Figure S8. ^{13}C-NMR spectrum of 2-(1-benzoyl-4,5-dihydro-1*H*-imidazol-2-yl)phthalazin-1(2*H*)-one (ligand **L3**) registered in DMSO-d_6 (100 MHz) **7**

Figure S9. Mass spectrum of 2-(1-benzoyl-4,5-dihydro-1*H*-imidazol-2-yl)phthalazin-1(2*H*)-one (ligand **L3**) **7**

Figure S10. IR spectrum of dichloro[2-(4,5-dihydro-1*H*-imidazol-2-yl)phthalazin-1(2*H*)-imine]copper(II) (complex **C1**) **8**

Figure S11. IR spectrum of dichloro[2-(1-benzoyl-4,5-dihydro-1*H*-imidazol-2-yl)phthalazin-1(2*H*)-one]copper(II) (complex **C2**) **8**

2. X-ray crystallographic studies

Figure S12. CheckCIF/PLATON report of 2-(1-benzoyl-4,5-dihydro-1*H*-imidazol-2-yl)phthalazin-1(2*H*)-one (ligand **L3**) **9**

Figure S13. CheckCIF/PLATON report of dichloro[2-(1-benzoyl-4,5-dihydro-1*H*-imidazol-2-yl)phthalazin-1(2*H*)-one]copper(II) (complex **C2**) **10**

3. Antimicrobial studies

Table S1. Minimum inhibitory concentration (MIC) and minimum bactericidal concentration (MBC) [μg/mL] with the standard deviation (± SD) of the free ligands **L1**, **L3** and their copper(II) complexes **C1**, **C2** on reference strains of bacteria and yeasts **11**

4. Calculated ADME/drug-likeness profiles

Table S2. Predicted physicochemical, pharmacokinetic and drug-likeness properties of ligands **L1**, **L3** and their copper(II) complexes **C1**, **C2** **12**

Figure S1. IR spectrum of 2-(4,5-dihydro-1*H*-imidazol-2-yl)phthalazin-1(2*H*)-imine (ligand **L1**)*

* for NMR and MS spectra of **L1** see *Supplementary Materials* in reference **57** [Balewski, Ł.; *et. al.* A. synthesis and structure of novel hybrid compounds containing phthalazin-1(2*H*)-imine and 4,5-dihydro-1*H*-imidazole cores and their sulfonyl derivatives with potential biological activities. *Int. J. Mol. Sci.* **2024**, *25*, 11495, doi.org/10.3390/ijms252111495]

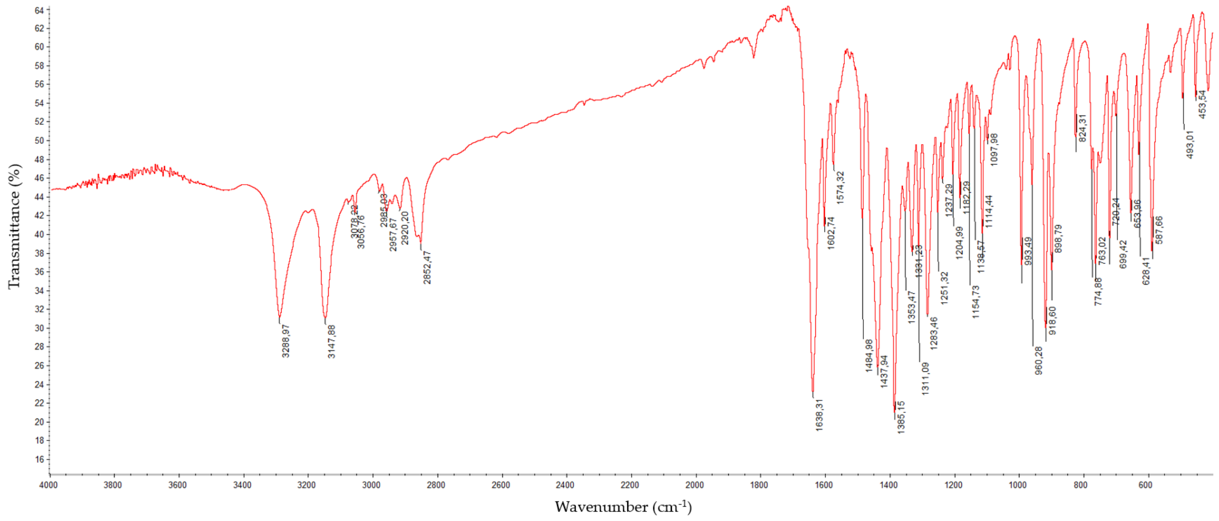

Figure S2. IR spectrum of *N*-(2-(1-benzoyl-4,5-dihydro-1*H*-imidazol-2-yl)phthalazin-1(2*H*)-ylidene)benzamide (ligand **L2**)

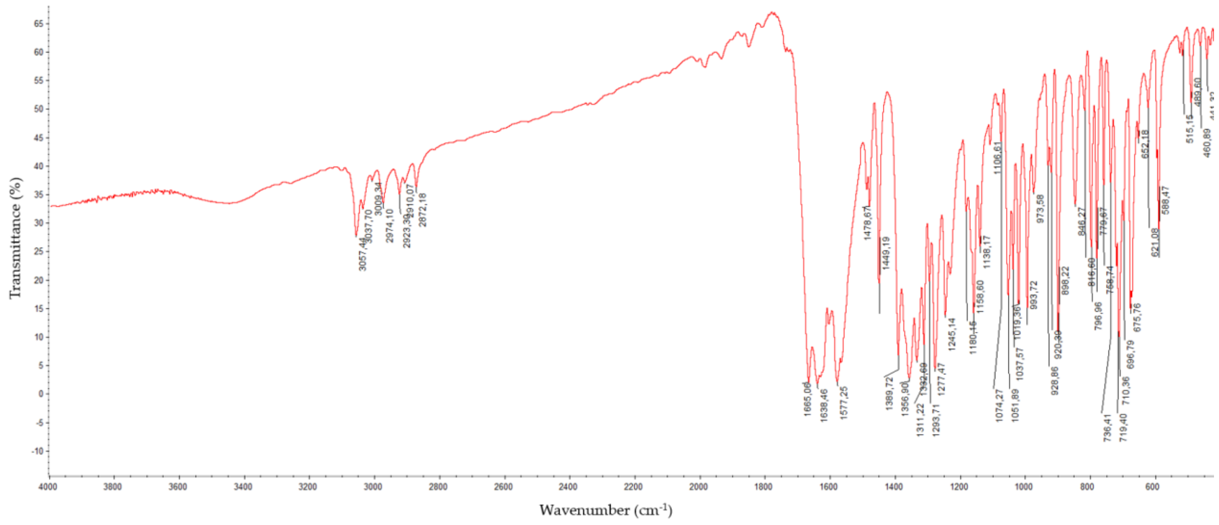

Figure S3. ^{1}H-NMR spectrum of *N*-(2-(1-benzoyl-4,5-dihydro-1*H*-imidazol-2-yl)phthalazin-1(2*H*)-ylidene)benzamide (ligand **L2**) registered in DMSO-d_6 (400 MHz)

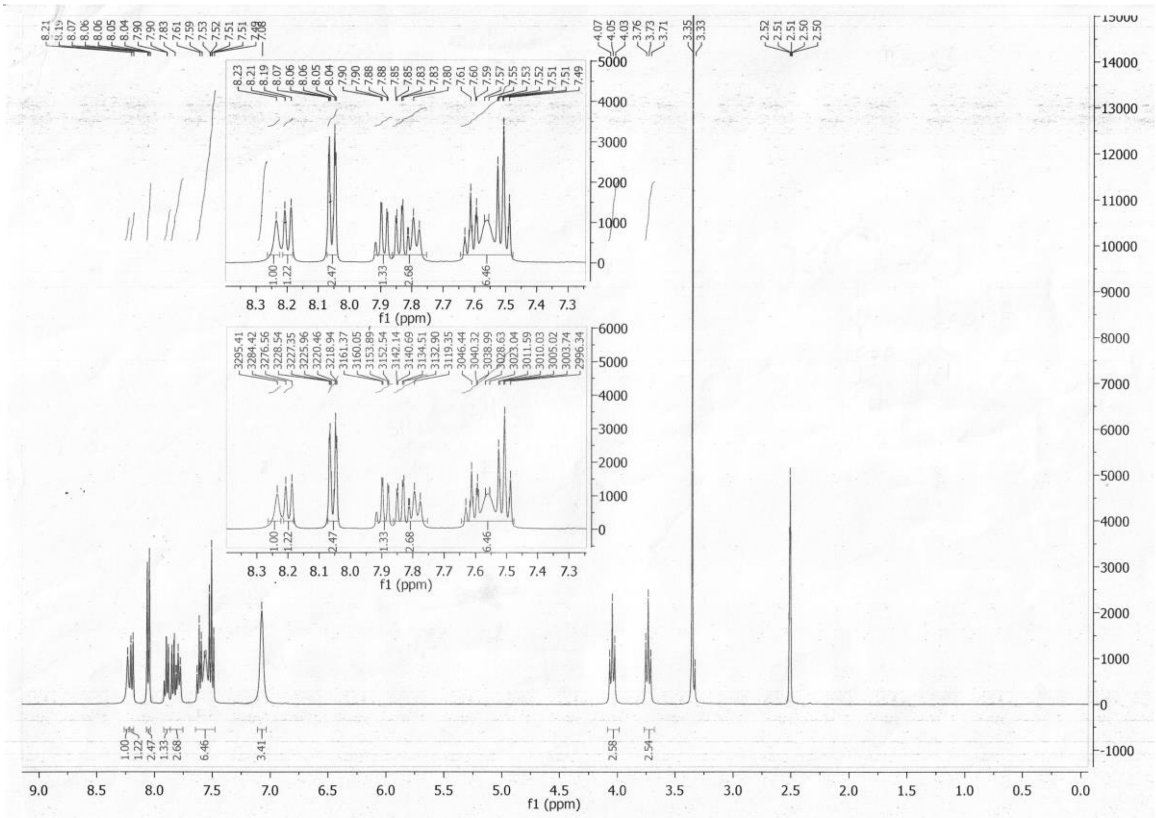

Figure S4. ^{13}C-NMR spectrum of *N*-(2-(1-benzoyl-4,5-dihydro-1*H*-imidazol-2-yl)phthalazin-1(2*H*)-ylidene)benzamide (ligand **L2**) registered in DMSO-d_6 (100 MHz)

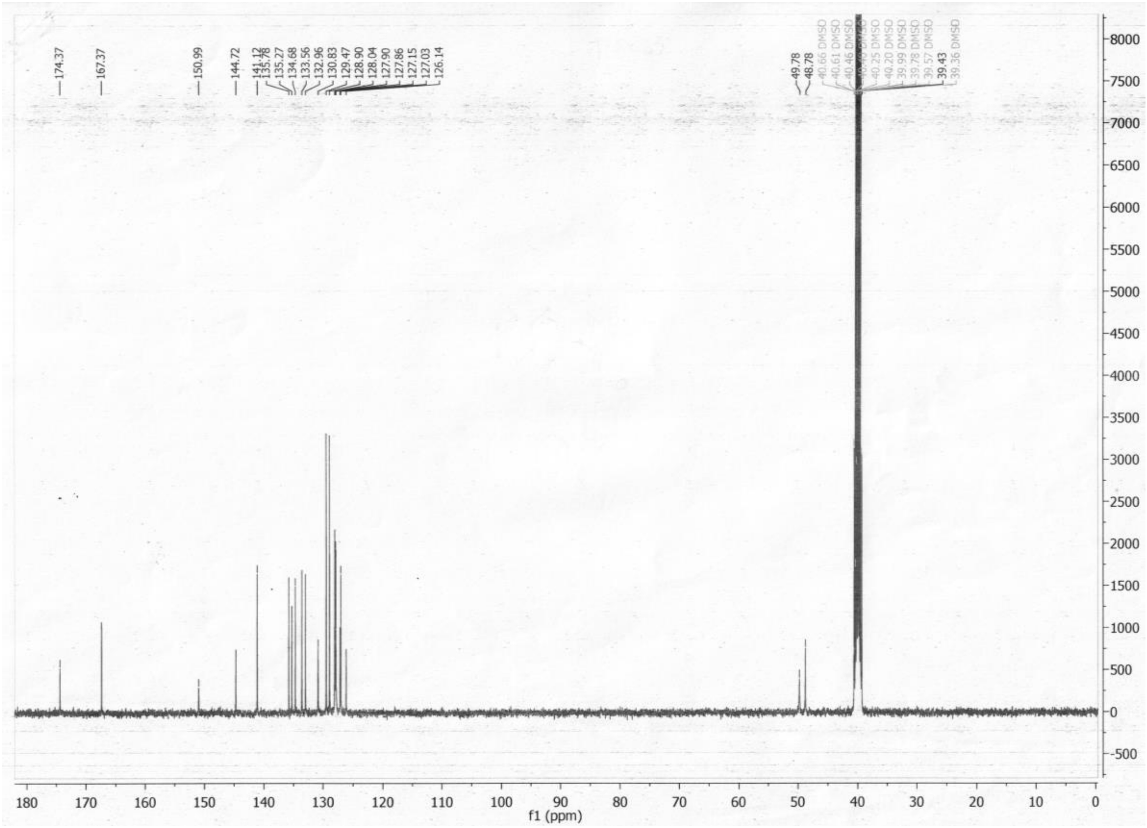

Figure S5. Mass spectrum of *N*-(2-(1-benzoyl-4,5-dihydro-1*H*-imidazol-2-yl)phthalazin-1(2*H*)-ylidene)benzamide (ligand **L2**)

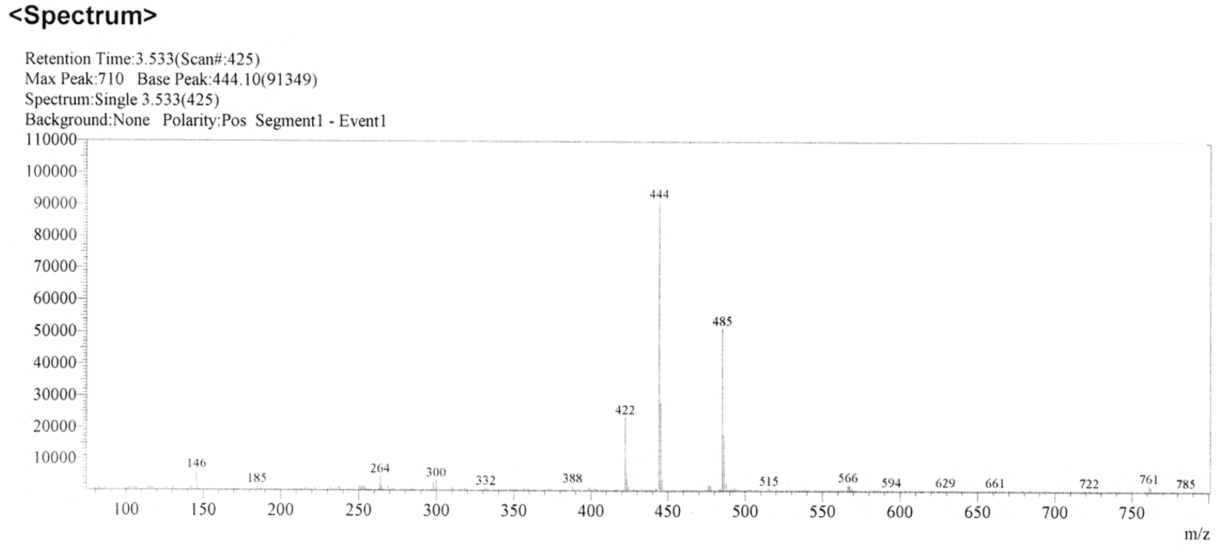

Figure S6. IR spectrum of 2-(1-benzoyl-4,5-dihydro-1*H*-imidazol-2-yl)phthalazin-1(2*H*)-one (ligand **L3**)

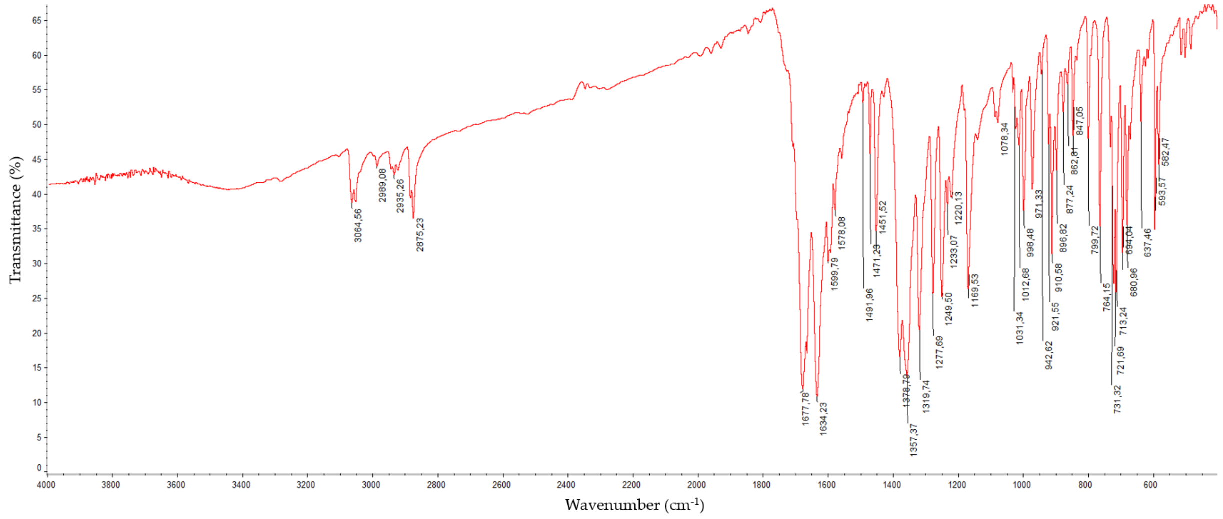

Figure S7. ^{1}H-NMR spectrum of 2-(1-benzoyl-4,5-dihydro-1*H*-imidazol-2-yl)phthalazin-1(2*H*)-one (ligand **L3**) registered in DMSO-d_6 (400 MHz)

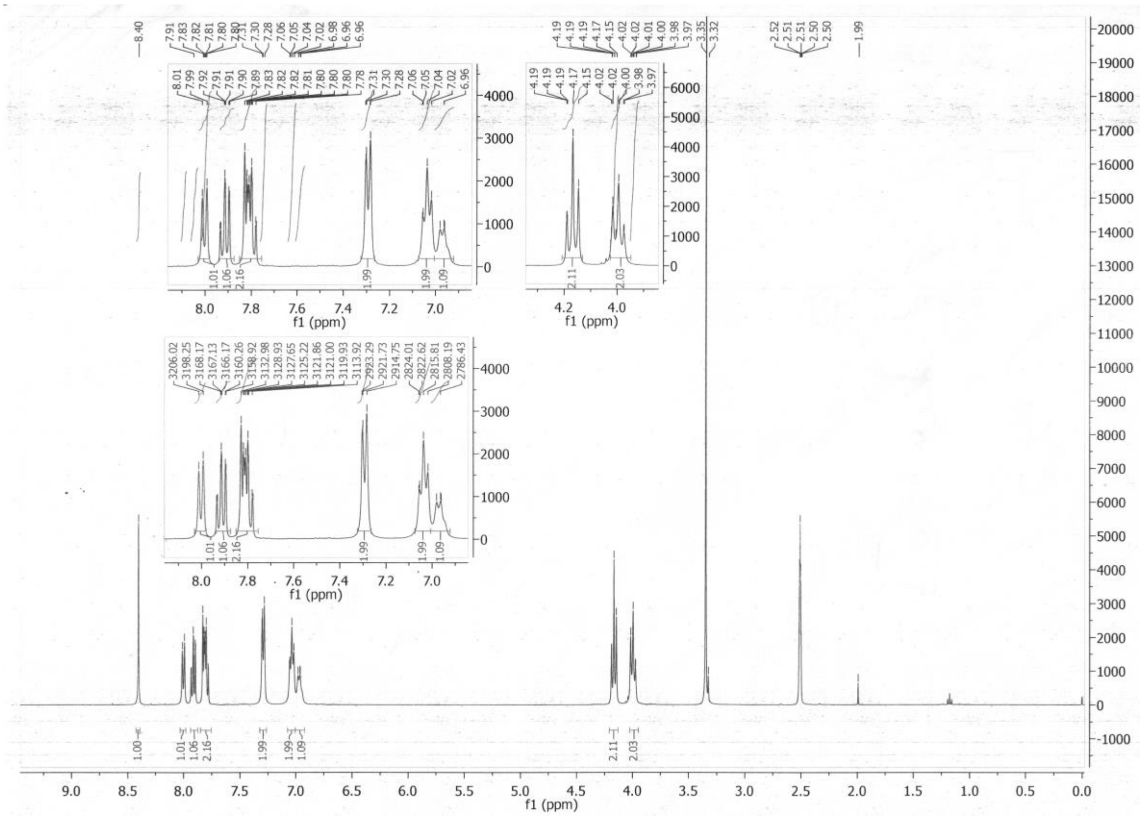

Figure S8. ^{13}C-NMR spectrum of 2-(1-benzoyl-4,5-dihydro-1*H*-imidazol-2-yl)phthalazin-1(2*H*)-one (ligand **L3**) registered in DMSO-d_6 (100 MHz)

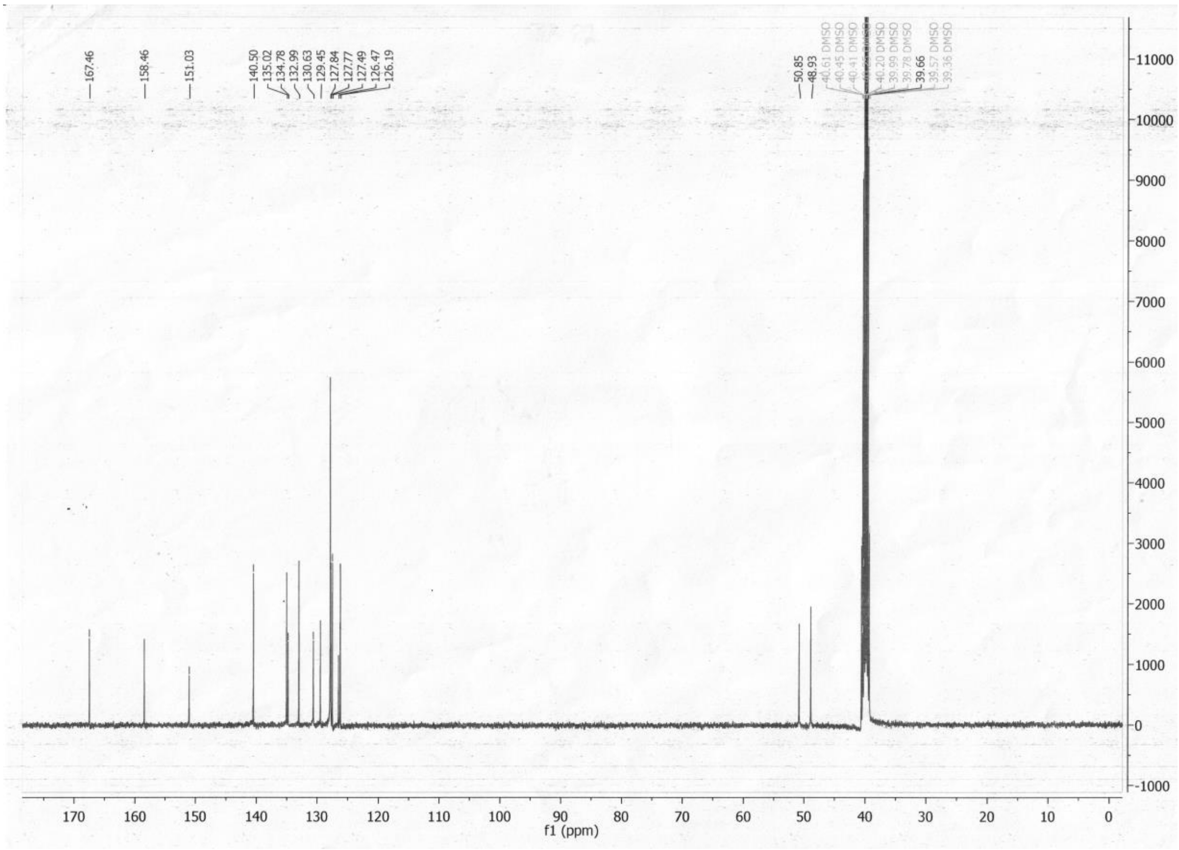

Figure S9. Mass spectrum of 2-(1-benzoyl-4,5-dihydro-1*H*-imidazol-2-yl)phthalazin-1(2*H*)-one (ligand **L3**)

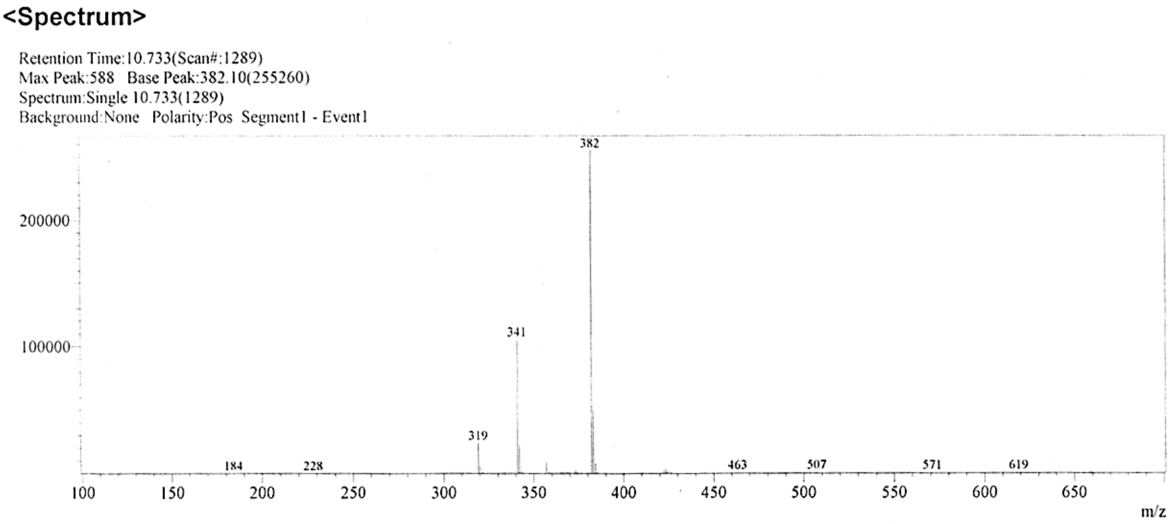

Figure S10. IR spectrum of dichloro[2-(4,5-dihydro-1*H*-imidazol-2-yl)phthalazin-1(2*H*)-imine]copper(II) (complex **C1**)

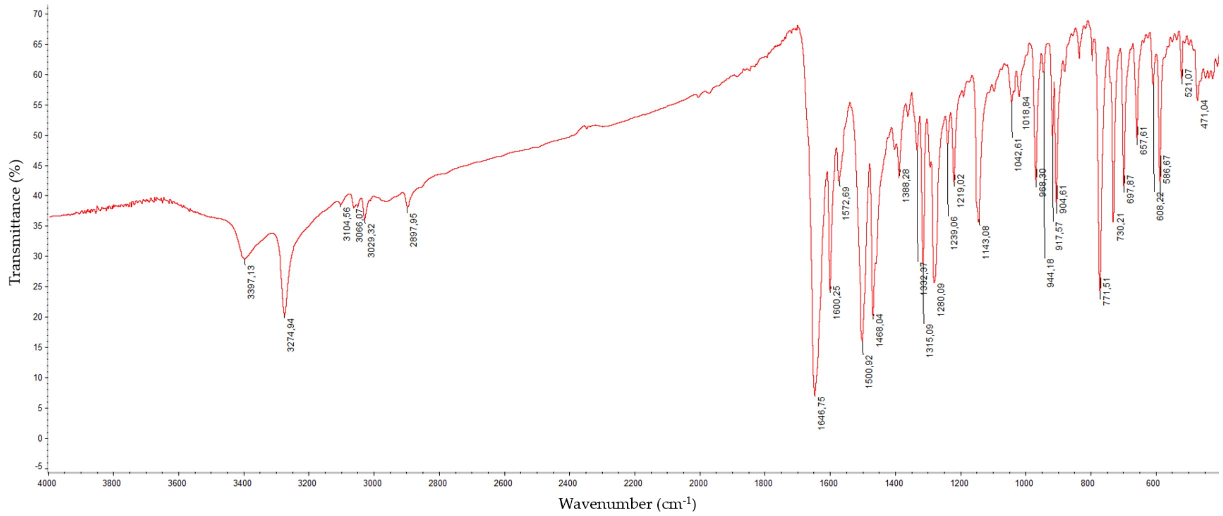

Figure S11. IR spectrum of dichloro[2-(1-benzoyl-4,5-dihydro-1*H*-imidazol-2-yl)phthalazin-1(2*H*)-one]copper(II) (complex **C2**)

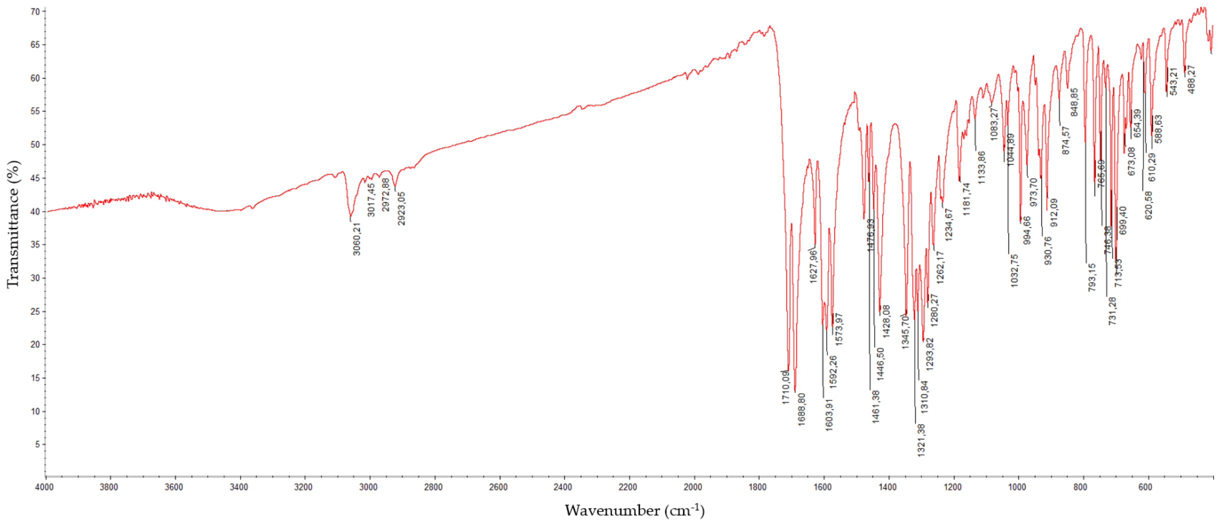

Figure S12. CheckCIF/PLATON report of 2-(1-benzoyl-4,5-dihydro-1*H*-imidazol-2-yl)phthalazin-1(2*H*)-one (ligand **L3**)

```
Bond precision:    C-C = 0.0021 A            Wavelength=0.71073

Cell:           a=8.3830(3)        b=8.7581(2)        c=10.8035(3)
                alpha=102.174(3)   beta=102.877(3)    gamma=91.121(2)
Temperature:    293 K

                 Calculated                  Reported
Volume           754.00(4)                   754.00(4)
Space group      P -1                        P -1
Hall group       -P 1                        -P 1
Moiety formula   C18 H14 N4 O2               C18 H14 N4 O2
Sum formula      C18 H14 N4 O2               C18 H14 N4 O2
Mr               318.33                      318.33
Dx,g cm-3        1.402                       1.402
Z                2                           2
Mu (mm-1)        0.095                       0.095
F000             332.0                       332.0
F000'            332.13
h,k,lmax         10,10,13                    10,10,13
Nref             3080                        3074
Tmin,Tmax        0.954,0.963                 0.974,1.000
Tmin'            0.954

Correction method= # Reported T Limits: Tmin=0.974 Tmax=1.000
AbsCorr = MULTI-SCAN

Data completeness= 0.998             Theta(max)= 26.371

R(reflections)= 0.0402( 2743)                          wR2(reflections)=
                                                       0.1009( 3074)
S = 1.036                  Npar= 217
```

Figure S13. CheckCIF/PLATON report of dichloro[2-(1-benzoyl-4,5-dihydro-1*H*-imidazol-2-yl)phthalazin-1(2*H*)-one]copper(II) (complex **C2**)

```
Bond precision:    C-C = 0.0028 A          Wavelength=1.54184

Cell:           a=7.38342(18)     b=10.0507(3)       c=12.8296(3)
                alpha=91.220(2)   beta=91.9414(18)   gamma=110.026(2)
Temperature:    293 K

                 Calculated                  Reported
Volume           893.44(4)                   893.44(4)
Space group      P -1                        P -1
Hall group       -P 1                        -P 1
Moiety formula   C36 H28 Cl4 Cu2 N8 O4       C18 H14 Cl2 Cu N4 O2
Sum formula      C36 H28 Cl4 Cu2 N8 O4       C18 H14 Cl2 Cu N4 O2
Mr               905.56                      452.77
Dx,g cm-3        1.683                       1.683
Z                1                           2
Mu (mm-1)        4.673                       4.673
F000             458.0                       458.0
F000'            456.54
h,k,lmax         9,12,16                     9,12,16
Nref             3760                        3714
Tmin,Tmax        0.453,0.598                 0.655,1.000
Tmin'            0.257

Correction method= # Reported T Limits: Tmin=0.655 Tmax=1.000
AbsCorr = MULTI-SCAN

Data completeness= 0.988           Theta(max)= 76.548

R(reflections)= 0.0266( 3480)                      wR2(reflections)=
                                                   0.0751( 3714)
S = 1.059                  Npar= 245
```

Table S1. Minimum inhibitory concentration (MIC) and minimum bactericidal concentration (MBC) [µg/mL] with the standard deviation (± SD) of the free ligands **L1**, **L3** and their copper(II) complexes **C1**, **C2** on reference strains of bacteria and yeasts.

**Microorganism / Compound**	**L1**	**C1**	**L3**	**C2**	Ciprofloxacin	Amphotericin B
			**MIC / MCB** [µg/mL]			
Gram-positive Bacteria						
S. aureus ATCC 6538	> 300	> 300	> 300	> 300	0.38 ± 0.1 / 0.58 ± 0.3	
Gram-negative Bacteria						
E. coli ATCC 8739	> 300	> 300	> 300	> 300	0.23 ± 0.08 / 0.84 ± 0.3	
Yeasts						
C. albicans ATCC 102231	> 300	> 300	> 300	> 300		0.5 ± 0.06 / 0.5 ± 0.08

Table S2. Predicted physicochemical, pharmacokinetic and drug-likeness properties of ligands **L1**, **L3** and their copper(II) complexes **C1**, **C2**

	Molecule	**L1**	**C1**	**L3**	**C2**
**Physico-chemical properties**	Formula	$C_{11}H_{11}N_5$	$C_{11}H_{11}Cl_2CuN_5$	$C_{18}H_{14}N_4O_2$	$C_{18}H_{14}Cl_2CuN_4O_2$
	MW	213.24	347.69	318.33	452.78
	Heavy atoms	16	19	24	27
	Aromatic heavy atoms	10	10	16	16
	Fraction Csp^3	0.18	0.18	0.11	0.11
	Rotatable bonds	1	1	3	3
	H-bond acceptors	3	3	4	4
	H-bond donors	2	2	0	0
	MR	68.75	80.45	98.30	90.30
	TPSA	66.06	66.06	67.56	67.56
**Lipophilicity**	iLOGP	1.84	0.00	2.38	0.00
	XLOGP3	0.27	1.87	1.80	3.41
	WLOGP	-0.44	0.94	1.00	2.37
	MLOGP	1.90	2.46	2.86	3.73
	Silicos-IT LogP	1.76	1.78	2.43	2.43
	Consensus LogP	1.07	1.41	2.09	2.39
**Water solubility**	ESOL Log S	-1.73	-3.50	-3.24	-5.04
	ESOL Solubility (mg/ml)	3.98e+00	1.11e-01	1.82e-01	4.17e-03
	ESOL Solubility (mol/l)	1.87e-02	3.18e-04	5.72e-04	9.20e-06
	ESOL Class	Very soluble	Soluble	Soluble	Moderately soluble
	Ali Log S	-1.22	-2.88	-2.84	-4.51
	Ali Solubility (mg/ml)	1.29e+01	4.59e-01	4.62e-01	1.40e-02
	Ali Solubility (mol/l)	6.04e-02	1.32e-03	1.45e-03	3.10e-05
	Ali Class	Very soluble	Soluble	Soluble	Moderately soluble
	Silicos-IT LogSw	-3.48	-3.48	-5.05	-5.05
	Silicos-IT Solubility (mg/ml)	7.13e-02	1.16e-01	2.80e-03	3.99e-03
	Silicos-IT Solubility (mol/l)	3.34e-04	3.34e-04	8.81e-06	8.81e-06
	Silicos-IT class	Soluble	Soluble	Moderately soluble	Moderately soluble
**Pharmaco-kinetics**	GI absorption	High	High	High	High
	BBB permeant	No	No	No	Yes
	P-gp substrate	No	No	No	No
	CYP1A2 inhibitor	Yes	Yes	Yes	No
	CYP2C19 inhibitor	No	No	Yes	Yes
	CYP2C9 inhibitor	No	No	No	No
	CYP2D6 inhibitor	No	No	No	No
	CYP3A4 inhibitor	No	No	No	No
	log Kp - skin permeation (cm/s)	-7.14	-7.09	-6.96	-5.64

Table S2. – cont.

**Drug-likeness**	Lipinski violations	0	0	0	0
	Ghose violations	1 (WLOGP <-0.4)	0	0	0
	Veber violations	0	0	0	0
	Egan violations	0	0	0	0
	Muegge violations	0	0	0	0
	Bioavailability Score	0.55	0.55	0.55	0.55
**Medicinal Chemistry**	PAINS alerts	0	0	0	0
	Brenk alerts	0	0	0	0
	Lead-likeness violations	1 (MW <250)	0	0	1 (MW >350)
	Synthetic Accessibility	2.98	3.12	3.19	3.33
